# Governance, coordination, and accountability in public health emergency preparedness and response: A scoping review

**DOI:** 10.1371/journal.pgph.0006719

**Published:** 2026-09-18

**Authors:** Sileshi Demelash Sasie, Melkamu Asrat Alava, Lensa Fekadu, Haymanot Firdie, Neima Zeynu Ali, Tarekegn Solomon, Melkamu Abte Afele

**Affiliations:** Ethiopian Public Health Institute, Addis Ababa, Ethiopia; PLOS: Public Library of Science, UNITED STATES OF AMERICA

## Abstract

Public health emergency preparedness depends not only on technical capacity but also on strong governance, coordinated multisector action, and clear accountability. However, evidence on how these systems function across different contexts remains fragmented. This scoping review synthesized global evidence on governance structures, coordination mechanisms, and accountability frameworks in public health emergency preparedness and response. Peer-reviewed and grey literature published between 2000 and 2025 was systematically searched in PubMed, Web of Science, Scopus, Embase, and CINAHL, as well as organizational and institutional websites. Eligible sources addressing governance, coordination, or accountability were charted and thematically analyzed to map institutional arrangements, coordination practices, and accountability systems. Seventy-seven sources met the inclusion criteria, comprising 67 primary studies and 10 grey-literature documents. Most studies were published after 2010, with strong representation from global, Asian, and African contexts. The evidence included conceptual and theoretical work, empirical studies, reviews and framework analyses, and policy-oriented guidance. Four interlinked themes emerged: governance architecture, legal foundations, and the distribution of authority, including centralized and decentralized arrangements; institutional roles and coordination mechanisms; accountability failures and system fragmentation; and reform approaches and enabling conditions. Clear legal mandates, functional intersectoral coordination bodies, and routine performance reviews were reported alongside stronger preparedness, whereas weak accountability, overlapping mandates, fragmented information systems, and political inertia were reported alongside delayed and poorly coordinated responses. Effective public health emergency preparedness was described in the literature less in relation to resources alone than to the strength and integration of governance, coordination, and accountability systems. Stronger preparedness was reported alongside adaptive governance, institutionalized multisector coordination, transparent oversight, and continuous learning embedded in policy and practice.

## Background

Effective coordination through multilateral frameworks, such as the International Health Regulations (IHR), is essential to strengthening global preparedness and response to public health emergencies [1]. Despite these efforts, establishing robust policies and partnerships that support accountable collaboration across nations and organizations remains a persistent challenge [2–8]. Under the IHR, State Parties are legally obligated to develop core oversight capacities for surveillance, investigation, and information sharing concerning cross-border health risks [1]. However, available evidence indicates that many countries including a substantial proportion of low- and middle-income States Parties that report weaker IHR legal and financing capacities in WHO State Party Self-Assessment Annual Reporting (SPAR) and Joint External Evaluation (JEE) assessments, for example settings such as Uganda, Bangladesh, and Pakistan described in the included literature have yet to fully implement the required legislative and administrative mechanisms, prompting renewed attention to policy reform and enforcement strategies [7,9,10].

The scale of governance required to mandate and monitor coordinated action across international networks of donors, non-governmental organizations (NGOs), humanitarian agencies, and national authorities is considerable [11–16]. Large-scale health emergencies such as the West African Ebola epidemic, the COVID-19 pandemic, cholera outbreaks linked to climate shocks, and cross-border influenza and mpox emergencies often emerge in multiple regions simultaneously and demand rapid mobilization of pooled resources, conditions under which decentralized accountability frameworks may falter. For instance, evaluations of emergency response programs have found that funding recipients sometimes divert allocated resources away from agreed objectives due to the absence of holistic performance monitoring frameworks between donors and implementing partners [11–14]. Transnational health crises, such as the Ebola epidemic and COVID-19 pandemics, have further exposed critical deficits in the verification of Member States’ compliance with surveillance, control, and information-sharing provisions outlined in accountability instruments like the IHR [1–4].

Regional coordination entities have increasingly sought to address governance gaps, albeit with mixed success. The Africa Centres for Disease Control and Prevention (Africa CDC) leads continent-wide initiatives to strengthen disease detection, preparedness, and cross-border policy harmonization [9,10,17,18]. Central to this regional architecture is the expanding model of National Public Health Institutes (NPHIs), promoted globally by the World Health Organization and the International Association of National Public Health Institutes as the core institutional platforms for surveillance, laboratory systems, emergency coordination, and evidence-to-policy translation [19–22]. However, despite the strategic positioning of both Africa CDC and NPHIs, these efforts are often constrained by financial limitations that hinder enforcement of recommended standards and the provision of sustained technical support to weaker health systems [19,23–25].

Ethiopia provides one illustrative national example of these dynamics. Federal responsibility for multisector coordination rests with the Ethiopian Public Health Institute (EPHI), which anchors national IHR implementation [18]; yet regional bureaus and woreda-level structures operate under heterogeneous disaster-operation guidelines and lack standardized reporting systems, and gaps in budget tracking, protocol adherence, inter-institutional corrective action, and digital-system interoperability have been reported [18,26–33]. The country’s COVID-19 response nonetheless demonstrated use of IHR core capacities and improved role clarity through regular drills, and its post-1990s shift from a reactive posture toward mitigation and preparedness supported by engagement with regional bodies such as IGAD marked a significant, if incomplete, transition [17,18,31,34,35]. These advances notwithstanding, enforcement, subnational implementation, and sustained technical support gaps remain, particularly in less-resourced regions [17,18,28,30,31].

Globally, more than 1,500 public health emergencies were reported annually between 2011 and 2018, underscoring the urgency of enhanced coordination and collective action [1,2]. While initiatives such as the Global Health Security Agenda have helped elevate political commitment, many preparedness objectives require deeper institutionalization and sustained investment [5,14–16]. This scoping review is designed to identify critical gaps and inconsistencies within current policy and institutional frameworks governing public health emergency preparedness and response across public health systems globally.

## Methods

### Study design

This scoping review was conducted in accordance with the methodological framework proposed by Arksey and O’Malley [36] and further refined by Levac et al. [37]. The review adhered to the PRISMA-ScR (Preferred Reporting Items for Systematic Reviews and Meta-Analyses extension for Scoping Reviews) checklist and was guided by the Joanna Briggs Institute methodology for scoping reviews [38,39]. The completed PRISMA-ScR checklist is provided in S1 PRISMA Checklist. The review aimed to map global evidence on policy and institutional arrangements that mandate and operationalize multisector accountability in emergency preparedness and response, including governance architecture, institutional mandates, coordination mechanisms, accountability arrangements, and reported gaps and inconsistencies. No protocol was registered.

### Research questions

The primary research question guiding this review was: What policy and institutional arrangements, coordination mechanisms, and accountability structures are reported in the literature as shaping multisector public health emergency preparedness and response? The review further addressed sub-questions on governance architectures and legal frameworks; institutional roles, mandates, and leadership arrangements; coordination mechanisms and operational structures; factors reported as influencing multisector preparedness; and gaps, fragmentation, and inconsistencies constraining coordination and accountability.

### Eligibility criteria

Sources were eligible if they examined policies, laws, plans, or institutional frameworks related to public health emergency preparedness and response; addressed multisector governance, coordination, accountability, or inter-agency arrangements; were relevant to national or subnational policy or institutional analysis; were peer-reviewed articles or grey-literature sources (policy documents, technical reports, evaluations, or institutional guidance); were available as full-text English-language publications; and were published between 1 January 2000 and 5 June 2025. Sources were excluded if they fell outside this window, did not address preparedness or multisector governance at the policy or institutional level, or were unavailable in full-text English.

### Information sources and search strategy

A structured and comprehensive search strategy was developed to identify relevant literature. Initial index terms and keywords were generated through exploratory searches (SDS, NZ, MA, HF) and iteratively refined. Systematic searches were conducted across PubMed, Scopus, Web of Science, CINAHL, and Embase, applied to titles, abstracts, and controlled-vocabulary fields, combining concepts related to emergency management, governance and policy frameworks, institutional arrangements, and accountability mechanisms using Boolean operators. Searches covered 1 January 2000–5 June 2025; all databases were last searched on 5 June 2025 and limited to English-language publications. Reference lists of included articles were screened to identify additional sources. The search strategy was independently reviewed for completeness and accuracy by a second reviewer before execution, consistent with peer review of electronic search strategies (PRESS). The full electronic search strategy for all five databases, including exact Boolean strings, field tags, limits, and the number of records retrieved per database, is presented in S1 Table.

### Study selection

**Fig 1:**
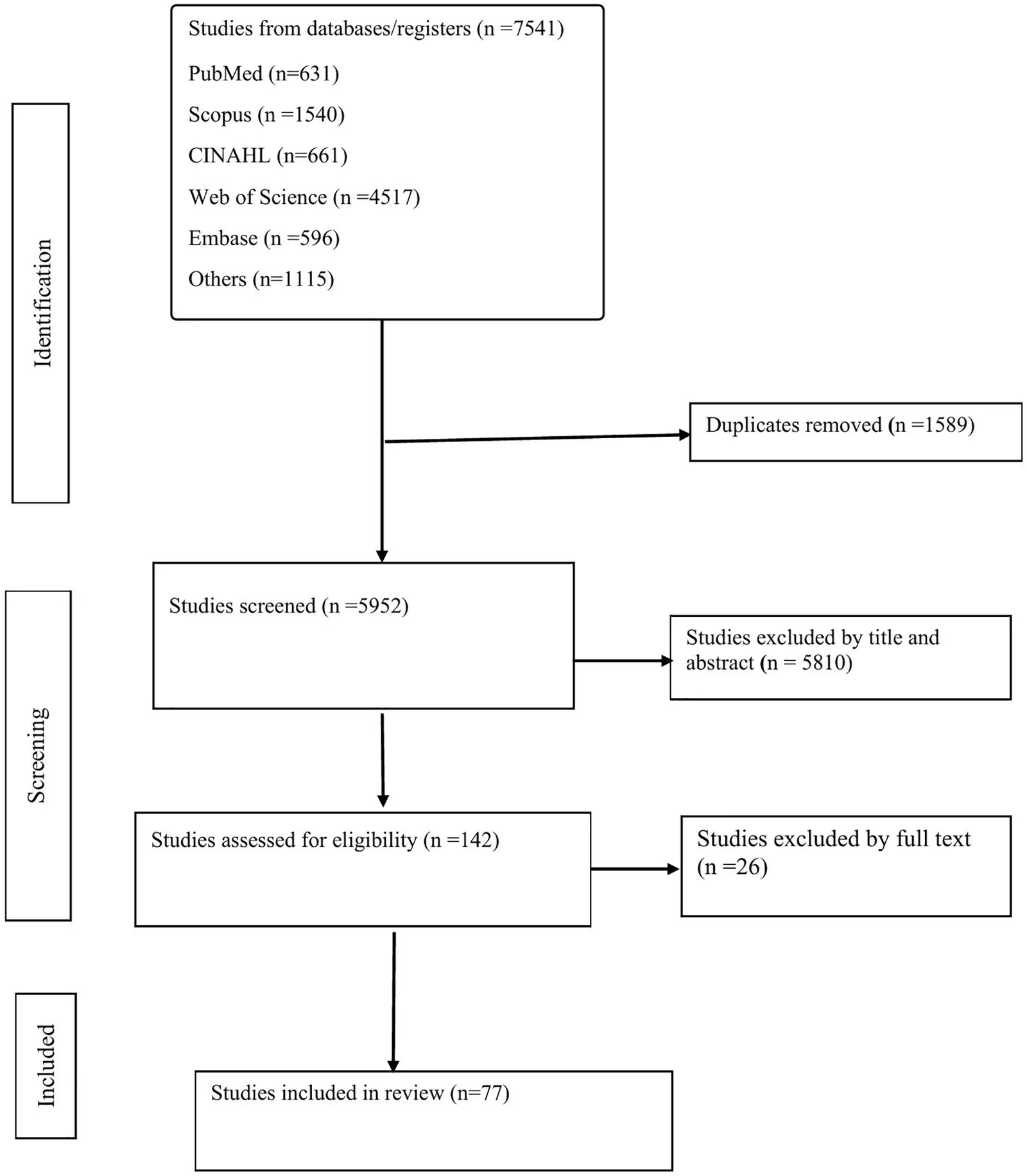
PRISMA-ScR flow diagram of the study selection process.

All retrieved records were imported into EndNote for de-duplication. Screening proceeded in two phases: title/abstract screening and full-text assessment, each conducted independently by two reviewers using predefined criteria. A calibration exercise on ten records yielded 90% raw agreement and a Cohen’s kappa of 0.82, indicating substantial inter-rater reliability [40]. Discrepancies were resolved through discussion and consensus, with adjudication by a third reviewer when necessary. The selection process is summarized in the PRISMA-ScR flow diagram (Fig 1).

### Data extraction

A standardized data-extraction framework was developed and piloted on a subset of studies, then applied to all included sources. The template captured bibliographic and contextual information, methodological characteristics, evidence type (empirical, conceptual/theoretical, review/framework, or grey-literature policy document), and reported elements including study-reported recommendations and limitations. Extraction was conducted independently by two reviewers (SDS and FMA), with discrepancies resolved by discussion and third-reviewer adjudication (DDA) where necessary. The complete data-extraction template, including all charted variables and their definitions, is provided in S2 Table.

### Data analysis

Analysis followed an inductive, descriptive approach consistent with scoping-review methodology. Extracted information was coded iteratively to organize reported content without imposing predefined analytical categories. The thematic analysis followed a six-phase process: (1) data familiarization; (2) initial code generation using descriptive labels; (3) code aggregation into candidate themes; (4) theme refinement against extracted data; (5) final theme definition and naming; and (6) identification of cross-cutting patterns. Coding was conducted independently by two reviewers (SDS and TS), with discrepancies resolved by discussion and adjudication by a third reviewer (NZA). The full coding framework comprising all codes, definitions, the six-phase analytical process, candidate-to-final theme mapping, and cross-cutting analytical decisions is provided in S3 Table to support transparency and reproducibility. The analysis focused on organizing reported information to support structured presentation, without assessing causality, effectiveness, prioritization, or comparative performance.

### Critical appraisal

Consistent with the objectives of a scoping review, no formal risk-of-bias or methodological quality assessment was undertaken; the review aimed to map the scope and characteristics of available evidence rather than to evaluate internal validity. Where reported, study limitations were extracted and summarized to support transparent interpretation.

## Results

### Flow of search and study inclusion

The initial electronic search identified 631 records from PubMed, 1,540 from Scopus, and 4,517 from Web of Science. After removal of 1,589 duplicates, 5,952 citations were screened by title and abstract. Of these, 142 articles met the eligibility criteria and were retrieved for full-text review. Following full-text assessment, 77 sources were included in the final scoping review. The selection process is shown in Fig 1.

### Characteristics of included studies

This scoping review included 77 sources examining multisector emergency coordination, classified by geographic scope, publication period, and publication type. Sources with a global or multi-regional scope were most frequent, followed by studies from Asia and Africa, while studies from Europe, North America, and South America were less frequently represented. With respect to publication period, most sources were published between 2000 and 2025, with a comparatively larger share appearing after 2010 and a smaller number indexed for 2021–2024. The comparatively smaller number of sources indexed for 2021–2024 likely reflects database indexing and publication lag at the search date, the English-language restriction, and the exclusion of non-governance commentary, rather than a genuine decline in pandemic-era scholarship; this temporal pattern should therefore be interpreted with caution.

By evidence type, included sources comprised empirical studies, conceptual or theoretical analyses, reviews and framework analyses, and grey-literature policy and evaluation documents (n = 10). The evidence type of each source is indicated in S4 Table, allowing readers to distinguish patterns supported by empirical data from those resting on conceptual argument or policy documentation. The literature addressed different phases of emergency management (preparedness, response, recovery) and spanned administrative levels from supranational to subnational. A detailed breakdown of study characteristics is presented in S4 Table.

### Key themes in strengthening multisector accountability in emergencies

Synthesis of the included peer-reviewed literature identified four higher-order analytical themes describing how multisector accountability is structured, operationalized, constrained, and addressed within public health emergency preparedness and response systems: (i) governance architecture, legal foundations, and the distribution of authority (including centralized and decentralized arrangements); (ii) institutional roles and coordination mechanisms; (iii) accountability failures and system fragmentation; and (iv) reform approaches and enabling conditions as reported across studies.

#### Theme 1: Governance architecture, legal foundations, and the distribution of authority.

Across the reviewed studies, governance architecture and legal foundations were described in relation to the organization, authority, and scope of multisector accountability. Empirical analyses of national emergency policies in Europe and the Asia-Pacific identified health, civil protection, interior, and allied sectors as core actors, while documenting substantial variation in how intersectoral roles were structured [41,42]. For example, post-Hurricane Katrina analyses in the United States described how ambiguous role definition was reported alongside coordination breakdowns, while post-Ebola legislative reforms in Guinea were described as strengthening accountability mandates [43,44]. Legislative and policy-focused studies described how legal instruments specifying mandates, coordination authority, and enforcement provisions were incorporated into emergency governance following crises, with variation in implementation across settings [45,46]. Conceptual and framework analyses applied analytical models to assess governance quality, identifying recurring gaps in coordination, oversight, and resource flows [47,48]. Regional and cross-border analyses described governance approaches addressing transnational risks [7,49], and collectively the literature described governance systems varying in legal clarity, sectoral integration, and alignment with international norms [43,46,50–54].

Within this governance architecture, studies also described the distribution of authority along a continuum from centralized to decentralized arrangements. Centralized arrangements were described as featuring national-level leadership, consolidated decision-making, and centralized resource mobilization, while decentralized arrangements involved greater delegation to subnational and community actors [41,43,55–57]. Evidence from Ethiopia described decentralized governance during the COVID-19 response, including community-level mobilization in the Southern Nations, Nationalities, and Peoples’ Region [56], with comparable descriptions in nutrition and post-epidemic contexts in Burkina Faso and Guinea [44,45]. Studies from South Africa, Pakistan, and the Eastern Mediterranean described decentralized or partially decentralized arrangements accompanied by coordination, capacity, or information-flow challenges [50,55,58], whereas analyses from Europe and the Americas described centralized frameworks emphasizing national coherence alongside reported limitations in local responsiveness during emergencies such as Hurricane Katrina and the Zika outbreak [41,43,57,59]. Across regions, governance structure was presented as an organizing feature reported in relation to preparedness dynamics rather than as a uniformly advantageous or disadvantageous model [41,43–45,50,55–59].

#### Theme 2: Institutional roles and coordination mechanisms.

The literature consistently described institutional roles and coordination mechanisms as central components of multisector emergency preparedness and response. Institutional responsibilities commonly included preparedness planning, oversight, coordination, logistics, surveillance, and operational response, frequently supported by legal authority and formalized standard operating procedures [7,48,60–64]. For example, empirical analyses of coordination in the Philippines (Manila) described how vertical and horizontal accountability arrangements were combined across municipal and national levels [65]. Coordination mechanisms were described as operating vertically (escalation protocols engaging higher-level institutions when local capacity was exceeded) and horizontally (coordination among institutions at similar levels) [59,65–72]. Formal structures such as emergency operations centers and multisector committees were described across settings, alongside communication protocols and activation triggers [7,73]. Several studies documented training, capacity-building, and performance-monitoring processes [67,69,70], and information-sharing protocols, integrated contingency planning, and joint resource management as operational features [74–77]. Coordination arrangements were reported to evolve over time in response to emerging risks and lessons learned [42,78–80].

#### Theme 3: Accountability failures and system fragmentation.

Despite the presence of governance and coordination frameworks, many studies documented persistent accountability challenges linked to institutional fragmentation. Overlapping mandates, unclear lines of authority, and weak alignment across agencies were frequently reported, particularly during cross-jurisdictional and multisector responses [43,50,68,78], and were described in relation to inefficient resource deployment, delayed response timelines, and reduced coherence [7,61,69]. For example, an empirical disaster-accountability analysis in Bangladesh described how fragmented mandates and limited local-government capacity were reported alongside weaker accountability during emergencies [78]. Variation in monitoring and evaluation (M&E) approaches was recurrent: studies reported differences in indicator selection, limited standardization, and an emphasis on health-sector metrics that constrained system-wide assessment [41,44,45,81,82]. Several studies described misalignment between accountability expectations and the availability of technical, financial, or human-resource support, particularly in low-resource settings [42,55,56,74,83,84]. Weak integration of community-level accountability was also commonly reported: while top-down oversight was frequently codified, bottom-up feedback processes were inconsistently formalized [49,72,77], a gap described in relation to limited responsiveness to local needs and reduced local ownership [57,59,76,85–88].

#### Theme 4: Reform approaches and enabling conditions reported in the literature.

The included studies described a range of reform approaches and contextual conditions associated with multisector accountability arrangements. Reported approaches included national coordination bodies linking humanitarian and development functions, formalization of intersectoral roles, and alignment with disaster risk reduction frameworks such as the Sendai Framework [70,80,89]. For example, community-based feedback mechanisms in nutrition programming in Burkina Faso were described as accompanying improved information sharing after a coordinating framework was established [44]. Reform efforts were described alongside conditions such as standardized accountability indicators, institutionalized simulation exercises, and interoperable information systems [59,67,89,90]. Community-based accountability and participatory monitoring were described within specific programmatic areas, including nutrition and maternal health [44,91]. Conceptual studies outlined accountability constructs responsibility, answerability, and liability as analytical lenses [51,84], and studies examining coordination described reform contexts in which legal frameworks, institutional capacity, coordination mechanisms, and stakeholder engagement were addressed concurrently, though their form varied across settings [59,63,68,87,88,90].

### Findings from grey literature

Table 1 summarises the ten grey-literature documents only; these are reported separately from peer-reviewed studies to make the type and source of evidence explicit and to avoid treating non-peer-reviewed policy documents as equivalent to empirical research. These sources include global frameworks, technical guides, evaluation tools, mission reports, discussion papers, and policy briefs described institutional, legal, and policy arrangements related to multisector accountability. Across documents, governance architectures commonly delineated multisector roles and coordination structures, while accountability was operationalized through assessment tools, performance indicators, and review mechanisms aligned with international standards such as the IHR. Sources also documented recurring implementation challenges, including limitations in subnational coordination, inter-agency communication, enforcement of mandates, financing, and workforce capacity.

**Table 1:** Grey-literature sources (n = 10): governance and accountability features described and implementation gaps reported, presented separately from peer-reviewed evidence.

Source	Region type	Features described implementation gaps reported
WHO, 2008 [1]	Global-Framework	Legally binding health-security requirements; core-capacity definition; cross-sector coordination limited enforcement; resource constraints; variable national capacity
WHO, 2005 [92]	Global-Technical guide	PHEIC declaration procedures; role clarification; communication protocols weak accountability; gaps in local implementation
WHO, 2022 [1]	Global-Evaluation tool	Structured assessment across 19 technical areas; self/external evaluation self-report reliability; high resource demand; limited follow-up
WHO, 2017 [93]	Pakistan-Mission report	IHR capacity; surveillance and laboratory performance; workforce needs funding limits; inter-agency challenges; skill gaps
WHO, 2015 [94]	Global -Technical report	Monitoring framework with core indicators; standardized assessment guidance data-quality and resource constraints; inconsistent implementation
Carter, 2021 [95]	Global South- Technical report	Community engagement and inclusivity; attention to vulnerable populations limited resources; coordination and capacity gaps
Sassa et al., 2020 [96]	Global-Book	Disaster risk reduction and multi-hazard approaches; international partnerships; knowledge sharing limited implementation capacity; resource constraints
Lundsgaarde, 2013 [97]	Global-Discussion paper	Institutional frameworks for development cooperation; donor coordination; local ownership fragmented donor approaches; sustainability challenges
Boin et al., 2016 [98]	Global-Book	Political and institutional dimensions of crisis management; leadership and coordination structures political constraints; competing institutional interests
Lupel & Snyder, 2017 [99]	West Africa-Policy brief	UN-system coordination in Ebola; stakeholder engagement; local capacity system fragmentation; resource-mobilization and communication barriers

### Factors reported in the literature related to preparedness and multisector coordination

Across the included sources, a wide range of factors related to public health emergency preparedness and multisector coordination were described, spanning governance frameworks, institutional roles, coordination mechanisms, fragmentation and inconsistencies, policy-level challenges, and approaches to multisector coordination. In keeping with the objectives of a scoping review, these factors are presented as a descriptive mapping of how preparedness- and coordination-related elements are reported, without implying effectiveness, prioritization, or normative recommendations. To make each factor concrete, Table 4 now includes an illustrative, country-anchored example of how the factor is reported in practice. A detailed cross-study mapping of reported factors and associated references is presented in Table 2.

**Table 2:** Factors reported in the literature related to governance and multisector coordination in health emergencies, with illustrative examples.

Domain	Representative factor	Illustrative example (as reported)
Governance frameworks	Legal mandates to enforce compliance with international regulations	*IHR (2005) core-capacity obligations enacted in national public-health legislation*
	Oversight mechanisms for monitoring adherence	*JEE and SPAR review cycles used to monitor national capacity*
Institutional roles & capacities	Defined roles and responsibilities for agencies	*NPHI (e.g., EPHI) assigned statutory lead for multisector coordination*
	Dedicated emergency-management agencies	*National disaster/EOC structures convening health and allied sectors*
Coordination & accountability	Multisector coordination bodies	*National incident-management committees and EOCs activated during outbreaks*
	Integrated digital systems for real-time data sharing	*Interoperable surveillance/HMIS platforms linking subnational and national levels*
Policy challenges	Sustainable funding mechanisms	*Domestic budget lines for preparedness rather than donor-dependent financing*
	Enforcement of laws and compliance	*Statutory authority with budgetary control and time-bound corrective actions*

**Fig 2:**
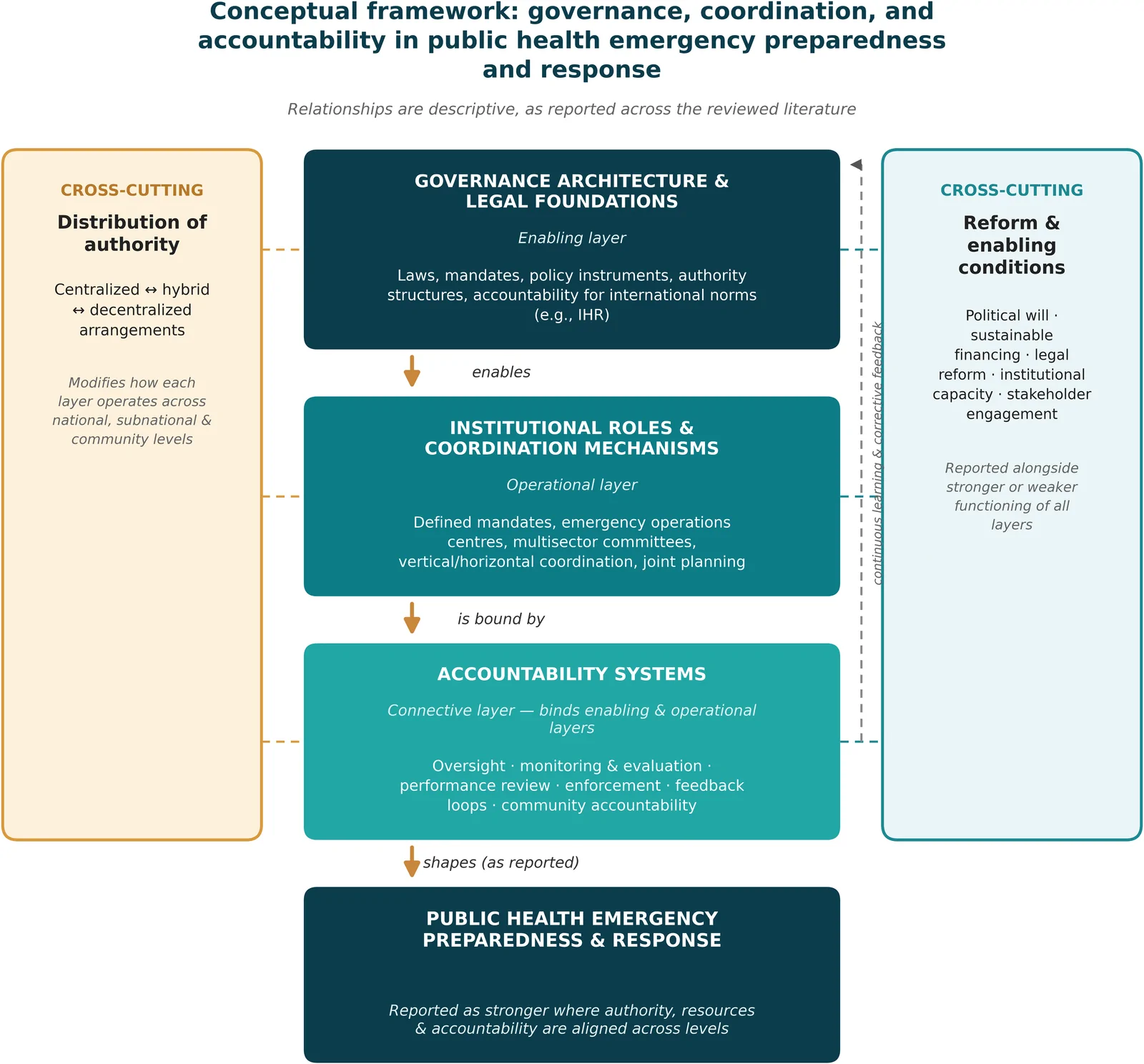
Conceptual framework integrating governance architecture, coordination mechanisms, and accountability systems in public health emergency preparedness and response.

The relationships among the governance, coordination, and accountability constructs mapped in this review are summarised descriptively in Fig 2. In this conceptual framework, governance architecture and legal foundations form the enabling layer; institutional roles and coordination mechanisms form the operational layer; accountability systems (oversight, monitoring, feedback, and community accountability) form the connective layer linking the other two; and reform/enabling conditions, together with the centralization–decentralization continuum, act as cross-cutting modifiers. Arrows denote relationships reported in the literature rather than demonstrated causal effects as shown in Fig 2 below.

## Discussion

This scoping review mapped how governance, coordination, and accountability in public health emergency preparedness and response are described across peer-reviewed and grey literature. Interpreted as a whole, the mapped evidence suggests that the recurring difficulty reported in the literature is one of translation from formally adopted policies and coordination structures into authority, resources, and accountability that operate in practice across institutional scales. This review adds to prior, more narrowly scoped reviews by mapping governance, coordination, and accountability as an interconnected system rather than as separate domains, by differentiating evidence types, and by offering an integrated descriptive framework.

Across the literature, formal legal and policy frameworks are widely reported, reflecting substantial alignment with international norms such as the IHR; however, the evidence consistently describes a gap between policy adoption and policy enforceability [100–107]. Interpreting this pattern, the literature frames preparedness weaknesses less as a deficit of policy instruments than as limitations in institutional authority, implementation capacity, and the accountability mechanisms that bind formal rules to practice. Closely linked is institutional fragmentation: overlapping mandates, unclear authority, and institutional competition are described as constraints on coordination [7,50,108–110]. Here the evidence is partly contradictory and warrants critical reading: while some preparedness frameworks treat additional coordination platforms as a remedy, the synthesised evidence suggests that, absent consolidated authority and aligned budgetary control, new bodies may reproduce rather than reduce fragmentation [7,108,111–114].

The literature also distinguishes structural from functional coordination: mechanisms such as emergency operations centers and incident-management systems are widely reported as formally established, yet their operational performance is described as uneven [7,115–117]. The interpretive implication is that coordination behaves as an institutional capacity built over time through authority, staffing, and financing, rather than as a discrete technical instrument created by policy designation [7,111,115,118]. Similarly, monitoring and evaluation outputs after-action reviews, Joint External Evaluations, national assessments are widely reported but often weakly connected to enforceable institutional change, functioning as reporting instruments rather than governing mechanisms [119–124].

A further recurring theme concerns the marginal role of community-level accountability within formal architectures. Despite strong rhetorical emphasis on community engagement, accountability arrangements are described as predominantly top-down and administrative [124–127], in contrast with the broader social-accountability literature documenting contributions of participatory monitoring to responsiveness and legitimacy [126,128–130].

Competing governance perspectives are evident in the evidence on centralization and decentralization. Comparative public-administration studies describe centralized arrangements as enabling unified command and rapid mobilization, yet also as constraining local responsiveness, whereas decentralized arrangements are described as improving contextual responsiveness yet also as fragmenting accountability where they are unfunded or lack technical capacity [131–136]. This does not imply that political leadership or financing are unimportant; on the contrary, the literature describes them as decisive enabling conditions. Rather, the evidence suggests that leadership and resources are reported to translate into preparedness most consistently when they are channeled through clear authority, defined mandates, and functioning accountability mechanisms, and least consistently where these governance arrangements are fragmented. On balance, the literature does not support either full centralization or full decentralization as universally superior; it more often describes hybrid configurations in which centralized authority is paired with institutionalized subnational capacity, financing, and enforceable accountability [131,132,136].

### Strengths and limitations

This review brings together a wide and diverse body of global evidence on multisector governance, coordination, and accountability, integrating empirical studies, conceptual analyses, and grey literature across regions. The use of PRISMA-ScR guidance and an inductive, layered thematic synthesis supported systematic interpretation, and the strong representation of low- and middle-income country evidence enhances relevance for African and similarly resourced settings.

Several limitations should be considered. Although multiple databases were searched, some relevant governance literature may not have been captured, and the English-language restriction may have excluded contributions from non–English-speaking regions. Grey-literature sources were included for their policy relevance but are not peer-reviewed and vary in methodological transparency; they were therefore synthesised and presented separately, interpreted with caution, and not weighted equally with peer-reviewed evidence. In addition, the review protocol was not registered, and because many included studies were descriptive and defined governance, coordination, and accountability heterogeneously, comparability across sources is limited and the mapped patterns should be read as descriptive associations rather than evaluative or causal findings. Finally, much of the included literature was descriptive, so publication bias and selective reporting cannot be excluded, and causal conclusions about effectiveness were beyond the scope of this review.

### Policy implications

Across the evidence, multisector emergency preparedness was most often reported in relation to whether legal authority, institutional mandates, and accountability mechanisms were enforceable in practice, rather than the mere presence of policies or coordination arrangements. Decentralization was reported alongside stronger preparedness mainly where it was matched with reliable financing, subnational technical capacity, and real decision-making authority. Community participation was reported as most consequential where it was formally built into accountability systems rather than treated as optional. Digital surveillance and coordination platforms were described as enabling when embedded within governance and oversight structures. Enforceability may be strengthened through mechanisms reported in the literature, such as statutory designation of a lead authority with budgetary control, binding inter-agency agreements specifying performance obligations, and the linkage of Joint External Evaluation and after-action-review findings to mandated, time-bound corrective actions subject to independent, publicly reported oversight approaches illustrated, for example, by the post-Ebola legislative assessment tool in Guinea and by accountability frameworks applied in polio eradication.

## Conclusion

This scoping review maps how multisector accountability in public health emergency preparedness and response is described in the literature in relation to how governance frameworks, legal authority, institutional mandates, coordination structures, and political–administrative conditions interact within health systems. Across diverse contexts, accountability was reported as strongest where roles and responsibilities were clearly defined in law, coordination mechanisms were institutionalized, and monitoring and feedback processes were actively used. By contrast, fragmented institutional mandates, weak enforcement of authority, limited subnational capacity, and minimal community-level accountability were consistently reported alongside weaker preparedness and response. The findings indicate that neither centralized nor decentralized governance is inherently superior; rather, accountability is described in relation to how well authority, resources, and information are aligned across levels. While global frameworks such as the IHR and JEE provide important reference points, their translation into enforceable national accountability arrangements remains uneven, particularly in low- and middle-income countries.

## Supporting information

S1 PRISMA Checklist Completed PRISMA-ScR checklist. (DOCX)

S1 Table Full database search strategies and retrieval counts. (DOCX)

S2 Table Data extraction template and variable definitions. (DOCX)

S3 Table Complete coding framework and codebook, including the six-phase thematic process, codes, definitions, and theme aggregation. (DOCX)

S4 Table Characteristics of included sources, including evidence-type classification. (DOCX)
