# Supplementary material for: Governance, coordination, and accountability in public health emergency preparedness and response: A scoping review
Source: PLOS Glob Public Health. 2026 Sep 18;6(9):e0006719. doi: 10.1371/journal.pgph.0006719 (PMC13600703; doi:10.1371/journal.pgph.0006719)
Supplement: Supplementary file 1 [file pgph.0006719.s007.pdf]

PLOS Global Public Health Governance, Coordination, and Accountability in Public Health Emergency Preparedness and Response: A Scoping Review

Manuscript Number PGPH-D-26-00735

Reviewer Comments

This is a strong and carefully conducted study that addresses an important gap in understanding governance arrangements for public health emergency preparedness and response. The analysis is well grounded in both empirical evidence and relevant frameworks, and the use of multiple data sources adds depth and credibility to the findings. The manuscript is timely, relevant, and offers practical insights for policymakers and health system actors. Overall, the paper is well written and makes a meaningful contribution. If the points outlined below are considered, the manuscript would be further strengthened and its clarity and overall impact improved.

1. Background

The background is comprehensive and clearly written. However, the section on Ethiopia appears relatively detailed compared to the broader global discussion. It may be helpful to present Ethiopia more briefly as an illustrative example or to streamline this section so that the global focus is maintained more consistently throughout.

2. Methods

The methods are appropriate and generally clear. It would be helpful to briefly clarify whether the search strategy itself was independently verified by a second reviewer, in addition to the screening process that is already well described.

3. Results

Theme 5 on centralization and decentralization appears to overlap with Theme 1 on governance architecture. It may be useful to further clarify the distinction between these themes or to consider merging them so that the structure of the results is easier to follow.

4. Limitations

The limitations are clearly presented and appropriate for a scoping review. It may, however, be useful to more explicitly reflect on how the descriptive nature of the included studies, together with variation in

how governance, coordination, and accountability are defined across contexts, may influence comparability of findings.

Overall Assessment

This is a well-prepared and thoughtful manuscript. With minor refinement, it is suitable for publication and will be useful for both national and global audiences working on public health emergency preparedness.

This is a solid and credible study with clear relevance to current global health priorities. The analysis is sound, and the findings are meaningful. I would recommend acceptance after minor revision.
