# Supplementary material for: Governance, coordination, and accountability in public health emergency preparedness and response: A scoping review
Source: PLOS Glob Public Health. 2026 Sep 18;6(9):e0006719. doi: 10.1371/journal.pgph.0006719 (PMC13600703; doi:10.1371/journal.pgph.0006719)
Supplement: Supplementary file 3 [file pgph.0006719.s002.docx]

S1 Table: Search Results by Database

| **Database** | **Search Terms** | **Number of Studies** |
| --- | --- | --- |
| PubMed | (("emergency management"[Title/Abstract] OR "emergency response"[Title/Abstract] OR "disaster*"[Title/Abstract] OR "crisis coordination"[Title/Abstract]) AND ("policy"[Title/Abstract] OR "governance"[Title/Abstract] OR "institution*"[Title/Abstract] OR "accountability"[Title/Abstract]) AND ("framework*"[Title/Abstract] OR "structure*"[Title/Abstract] OR "mechanism*"[Title/Abstract] OR "coordination"[Title/Abstract] OR "collaboration"[Title/Abstract])) AND ((ffrft[Filter]) AND (fha[Filter]) AND (humans[Filter]) AND (english[Filter]) AND (2000:2025[pdat])) | 631 |
| Web of Science | TS=(("emergency management" OR "emergency response" OR disaster* OR "crisis coordination") AND (policy OR governance OR institution* OR accountability) AND (framework* OR structure* OR mechanism* OR coordination OR collaboration)) | 4,517 |
| Scopus | (("emergency management" OR "emergency response" OR disaster* OR "crisis coordination") AND (policy OR governance OR institution* OR accountability) AND (framework* OR structure* OR mechanism* OR coordination OR collaboration)) | 1,540 |
| CINHAL | TI OR AB ("emergency management" OR "emergency response" OR disaster* OR "crisis coordination") AND TI OR AB (policy OR governance OR institution* OR accountability) AND TI OR AB (framework* OR structure* OR mechanism* OR coordination OR collaboration) | 661 |
| Embase | (title,abstract:("emergency management" OR "emergency response" OR disaster* OR "crisis coordination")) AND (title,abstract:(policy OR governance OR institution* OR accountability)) AND (title,abstract:(framework* OR structure* OR mechanism* OR coordination OR collaboration)) | 596 |
