# Supplementary material for: Governance, coordination, and accountability in public health emergency preparedness and response: A scoping review
Source: PLOS Glob Public Health. 2026 Sep 18;6(9):e0006719. doi: 10.1371/journal.pgph.0006719 (PMC13600703; doi:10.1371/journal.pgph.0006719)
Supplement: Supplementary file 4 [file pgph.0006719.s003.docx]

S2 Table: Data extraction template

**Section 1 · Bibliographic and contextual variables**

| **Variable** | **Definition / what was charted** | **Format / response options** |
| --- | --- | --- |
| **Source ID** | Unique identifier assigned to each included source for tracking across screening, extraction, and synthesis. | *Free text (e.g., S001)* |
| **Author(s)** | First author and co-authors, or issuing organization for grey literature. | *Free text* |
| **Year of publication** | Calendar year the source was published or released. | *Year (2000–2025)* |
| **Title** | Full title of the article, report, or document. | *Free text* |
| **Source / journal or publisher** | Journal name (peer-reviewed) or issuing body/repository (grey literature). | *Free text* |
| **Country / region** | Geographic focus of the source. | *Country; or region; or “global/multi-regional”* |
| **Income classification** | World Bank income grouping of the setting examined, where applicable. | *LIC / LMIC / UMIC / HIC / mixed / NA* |
| **Emergency context** | Type of emergency or hazard addressed (if specified). | *Outbreak / pandemic / disaster / multi-hazard / generic / NA* |
| **Emergency phase** | Phase(s) of emergency management addressed. | *Preparedness / response / recovery / cross-phase* |
| **Administrative level** | System level(s) addressed by the source. | *Supranational / national / subnational / community / multiple* |

**Section 2 · Methodological and evidence-type variables**

| **Variable** | **Definition / what was charted** | **Format / response options** |
| --- | --- | --- |
| **Evidence type** | Classification of the source by the kind of evidence it provides. Added at revision to support evidence differentiation across the synthesis. | *Empirical / conceptual–theoretical / review–framework / grey-literature policy* |
| **Study / document design** | Specific design or document category as reported by the source. | *e.g., systematic review, qualitative, mixed-methods, commentary, framework, mission report* |
| **Data source(s)** | Nature of the underlying data or basis of the source, where reported. | *Primary data / secondary / document analysis / expert opinion / NA* |
| **Peer-review status** | Whether the source is peer-reviewed or grey literature. | *Peer-reviewed / grey literature* |
| **Reported limitations** | Limitations explicitly stated by the source authors (charted verbatim or summarized). | *Free text* |

**Section 3 · Governance, coordination, and accountability content**

*These variables charted the substantive content relevant to the review questions. Each was recorded descriptively (present/reported, with a short supporting note) rather than scored or rated.*

| **Variable** | **Definition / what was charted** | **Format / response options** |
| --- | --- | --- |
| **Governance architecture & legal foundations** | Formal rules, laws, policy instruments, mandates, and authority structures organizing preparedness and response (Theme 1). | *Reported (Y/N) + note* |
| **Distribution of authority** | Centralized, decentralized, or hybrid arrangements described, and how authority/decision-making is distributed across levels (Theme 1 sub-element). | *Centralized / decentralized / hybrid / NA + note* |
| **Institutional roles & mandates** | Assignment of responsibilities to specific organizations (e.g., NPHI, EOC, sectoral ministries). | *Reported (Y/N) + note* |
| **Coordination mechanisms** | Structures and processes for inter-agency collaboration (EOCs, committees, escalation/activation protocols, information sharing, joint planning) (Theme 2). | *Reported (Y/N) + note* |
| **Accountability arrangements** | Oversight, monitoring and evaluation, performance review, enforcement, and feedback mechanisms, including community accountability (Themes 2–3). | *Reported (Y/N) + note* |
| **Fragmentation / gaps** | Overlapping mandates, unclear authority, weak enforcement, information-system incompatibility, or other reported breakdowns (Theme 3). | *Reported (Y/N) + note* |
| **Reform approaches & enabling conditions** | Strategies, recommendations, and contextual enablers for strengthening accountability and coordination (Theme 4). | *Reported (Y/N) + note* |
| **Reported factors** | Specific preparedness/coordination factors named by the source (mapped to the factor framework, Table 4). | *Free text / coded labels* |
| **Author recommendations** | Recommendations made by the source for policy or institutional strengthening. | *Free text* |
| **Illustrative country example** | Concrete national/subnational instance described, used to anchor thematic examples at revision. | *Free text / NA* |

**Notes on use**

- All variables were charted descriptively to map the breadth and nature of the evidence; no variable was used to grade quality, infer effectiveness, or rank sources.
- The “evidence type” and “illustrative country example” variables were added at revision in response to reviewer and editor comments on evidence differentiation and concreteness; all other variables were applied throughout the original extraction.
- The completed extraction matrix for all 77 included sources is provided as S4 Table (study characteristics). The coding framework derived from these variables is provided in S3 File.
