# Supplementary material for: Governance, coordination, and accountability in public health emergency preparedness and response: A scoping review
Source: PLOS Glob Public Health. 2026 Sep 18;6(9):e0006719. doi: 10.1371/journal.pgph.0006719 (PMC13600703; doi:10.1371/journal.pgph.0006719)
Supplement: Supplementary file 5 [file pgph.0006719.s004.docx]

S3 Table: Complete coding framework

| **Phase** | **Step** | **Code / Theme** | **Definition** |
| --- | --- | --- | --- |
| **Phase 1** | Data familiarization | All 77 included sources | Complete reading of all sources; key passages related to governance, coordination, and accountability highlighted |
| **Phase 2** | Initial code generation | LEGAL_FRAMEWORK | Mention of laws, regulations, or legally binding instruments |
|  |  | POLICY_GUIDELINE | Mention of formal policy documents, plans, or guidelines |
|  |  | LEGAL_MANDATE | Legal authority to enforce compliance with regulations |
|  |  | OVERSIGHT_MECHANISM | Systems for monitoring adherence to rules or standards |
|  |  | CRISIS_PROTOCOL | Formal procedures for managing public health emergencies |
|  |  | SECURITY_INTEGRATION | Linking public health policy with national security frameworks |
|  |  | ROLE_DEFINED | Clear specification of institutional responsibilities |
|  |  | ROLE_AMBIGUITY | Unclear, overlapping, or competing institutional mandates |
|  |  | CAPACITY_BUILDING | Programs to develop personnel skills or institutional capabilities |
|  |  | INTER_AGENCY_COLLABORATION | Mechanisms for cooperation across different agencies |
|  |  | DEDICATED_AGENCY | Establishment of specific emergency management organizations |
|  |  | SIMULATION_TRAINING | Regular drills or exercises to test readiness |
|  |  | COORDINATION_BODY | Formal multisectoral coordination structures |
|  |  | COMMUNICATION_FRAMEWORK | Systems or protocols for information exchange |
|  |  | JOINT_PLANNING | Collaborative planning processes across stakeholders |
|  |  | DIGITAL_SYSTEM | Integrated technology platforms for real-time data sharing |
|  |  | RESOURCE_MOBILIZATION | Mechanisms for pooling or allocating resources across sectors |
|  |  | EVALUATION | Regular assessments to identify policy or system weaknesses |
|  |  | STAKEHOLDER_ENGAGEMENT | Inclusion of local perspectives in decision-making |
|  |  | EQUITABLE_RESOURCE_ALLOCATION | Fair distribution of resources for preparedness |
|  |  | FRAMEWORK_ASSESSMENT | Systematic review of existing legal and institutional arrangements |
|  |  | LESSONS_LEARNED | Documentation of best practices from past events |
|  |  | ENFORCEMENT_STRATEGY | Methods to ensure compliance with laws or policies |
|  |  | POLITICAL_WILL | Visible commitment from political leadership |
|  |  | SUSTAINABLE_FUNDING | Long-term, reliable financing mechanisms |
|  |  | BUREAUCRATIC_BARRIER | Administrative obstacles to policy implementation |
|  |  | PUBLIC_AWARENESS | Community knowledge and support for preparedness |
|  |  | INSTITUTIONAL_REFORM | Recommended changes to organizational structures |
|  |  | COMMUNITY_PARTICIPATION | Community involvement in planning and decision-making |
|  |  | INNOVATIVE_TECHNOLOGY | Use of new technologies for coordination |
|  |  | FEEDBACK_MECHANISM | Systems for continuous improvement through feedback |
|  |  | CROSS_SECTOR_FRAMEWORK | Integrated accountability across multiple sectors |
|  |  | EVIDENCE_BASED_PRACTICE | Implementation of approaches supported by research |
|  |  | CONTEXTUAL_ADAPTATION | Tailoring strategies to local conditions |
|  |  | M_AND_E_METRICS | Indicators and tools for monitoring and evaluation |
|  |  | PHASED_IMPLEMENTATION | Gradual, staged approach to implementation |
|  |  | CENTRALIZED | Authority concentrated at national level |
|  |  | DECENTRALIZED | Authority delegated to subnational levels |
| **Phase 3** | Code aggregation | **Candidate Theme 1:** Governance architecture and legal foundations | LEGAL_FRAMEWORK + POLICY_GUIDELINE + LEGAL_MANDATE + OVERSIGHT_MECHANISM + CRISIS_PROTOCOL + SECURITY_INTEGRATION + CENTRALIZED + DECENTRALIZED |
|  |  | **Candidate Theme 2:** Institutional roles and coordination mechanisms | ROLE_DEFINED + ROLE_AMBIGUITY + INTER_AGENCY_COLLABORATION + DEDICATED_AGENCY + COORDINATION_BODY + COMMUNICATION_FRAMEWORK + JOINT_PLANNING + DIGITAL_SYSTEM + RESOURCE_MOBILIZATION + SIMULATION_TRAINING + CAPACITY_BUILDING |
|  |  | **Candidate Theme 3:** Accountability failures and system fragmentation | EVALUATION + STAKEHOLDER_ENGAGEMENT + EQUITABLE_RESOURCE_ALLOCATION + FRAMEWORK_ASSESSMENT + LESSONS_LEARNED + ENFORCEMENT_STRATEGY + BUREAUCRATIC_BARRIER + PUBLIC_AWARENESS |
|  |  | **Candidate Theme 4:** Reform approaches and enabling conditions | INSTITUTIONAL_REFORM + COMMUNITY_PARTICIPATION + INNOVATIVE_TECHNOLOGY + FEEDBACK_MECHANISM + CROSS_SECTOR_FRAMEWORK + EVIDENCE_BASED_PRACTICE + CONTEXTUAL_ADAPTATION + M_AND_E_METRICS + PHASED_IMPLEMENTATION + POLITICAL_WILL + SUSTAINABLE_FUNDING |
| **Phase 4** | Theme refinement | **Theme 1:** Governance architecture and legal foundations | Kept distinct – addresses formal rules, laws, and authority distribution |
|  |  | **Theme 2:** Institutional roles and coordination mechanisms | Kept distinct – addresses who does what and how they work together |
|  |  | **Theme 3:** Accountability failures and system fragmentation | Kept distinct – addresses barriers, gaps, and breakdowns |
|  |  | **Theme 4:** Reform approaches and enabling conditions | Kept distinct – addresses solutions and enabling factors |
|  |  | Centralization and decentralization | Integrated into Theme 1 as a sub-theme |
| **Phase 5** | Final theme definition | **Theme 1:** Governance architecture and legal foundations | The formal and informal rules, legal instruments, and power structures that organize emergency preparedness and response |
|  |  | **Theme 2:** Institutional roles and coordination mechanisms | The assignment of responsibilities to specific organizations and the structures enabling collaboration |
|  |  | **Theme 3:** Accountability failures and system fragmentation | Breakdowns in oversight, enforcement, and integration that undermine emergency response |
|  |  | **Theme 4:** Reform approaches and enabling conditions | Strategies, recommendations, and contextual factors that strengthen accountability and coordination |
| **Phase 6** | Cross-cutting patterns | Policy adoption versus enforcement gap | Legal frameworks exist but lack implementation capacity, resources, or enforcement mechanisms |
|  |  | Structural versus functional coordination | Coordination bodies exist (structural) but lack authority, staffing, or budget to function effectively (functional) |
|  |  | Centralization-decentralization trade-offs | Centralized systems enable unified command but may ignore local needs; decentralized systems enable responsiveness but risk fragmentation |
|  |  | Community accountability gap | Accountability arrangements remain top-down and administrative; community feedback mechanisms are absent or informal |
