# Supplementary material for: Governance, coordination, and accountability in public health emergency preparedness and response: A scoping review
Source: PLOS Glob Public Health. 2026 Sep 18;6(9):e0006719. doi: 10.1371/journal.pgph.0006719 (PMC13600703; doi:10.1371/journal.pgph.0006719)
Supplement: Supplementary file 6 [file pgph.0006719.s005.docx]

**S4 Table:** Characteristics of Studies in the Comprehensive Analysis of Accountability Frameworks in Emergency preparedness and response

| **Authors, Year** | **Continent, Country** | **Design** | **Pertinent Findings** | **Recommendations** | **Limitations** |
| --- | --- | --- | --- | --- | --- |
| Kamga et al., 2022 | Europe, Multiple countries | Systematic Review | Identified key sectors involved in emergency preparedness and response: health, civil protection, interior ministries. Found multi sectoral coordination and clearly defined roles/responsibilities across domains are critical for an effective response. However, approaches varied considerably between countries. | Develop clear guidance and standard operating procedures around sector roles, responsibilities, and coordination mechanisms. | Review scope was limited to Europe so findings may not be generalizable globally. |
| Koliba et al., 2011 | North America, United States | Qualitative | Post Hurricane Katrina assessment revealed a lack of clearly established accountability relationships and overlap/ambiguity between organizations involved in response led to coordination issues. Confusion around roles and responsibilities hampered efforts. | Recommendations focused on pre-event planning to clearly define roles for all stakeholders and ensure coordinated preparedness activities. | Analyzed a single event so may not reflect all situations. |
| Diasso et al., 2020 | Africa, Burkina Faso | Mixed methods | Evaluation of nutrition data systems found accountability and information sharing improved after coordinating framework was established between sectors. However, continued information gaps at peripheral levels were identified. | Proposed formalizing community feedback mechanisms and investing in digital technologies to strengthen multidirectional information flows. | Findings may not be generalizable beyond the specific national context. |
| El Jardali et al., 2024 | Asia | Review | Review of COVID 19 responses highlighted opportunities for enhanced multi sector collaboration in areas like surveillance, risk communication and logistics management. Key enablers included strong political leadership and preexisting partnerships. | Recommended institutionalizing preparedness networks that bridge sectors and developing structures to facilitate cooperative arrangements. | Generalizability limited since multi country analysis was not conducted. |
| Hall et al., 2019 | Global | Methodological | Developed framework to systematically assess non-traditional disaster response literature such as reports and guidelines. Provides criteria for extracting key elements of interventions described. | Framework allows diverse evidence to be systematically appraised to draw lessons for preparedness/response. | Framework was not empirically tested on large scale. |
| Attal Juncqua et al., 2019 | Africa, Guinea | Qualitative | Legislative assessment tool developed post Ebola was found to strengthen health security capacities and accountability mechanisms. | Recommended institutionalizing use of tool for monitoring outcomes over time and driving continued quality improvement. | Pilot study conducted in single country only. |
| Vicario Merino et al., 2019 | Asia, Philippines | Mixed methods | National and sub national coordination and planning between health and other sectors found to be critical for robust multi hazard preparedness. | Proposed standardizing approaches across regions and deepening community participation. | Sub national study conducted, so generalizability limited. |
| Bovens, 2007 | Europe | Conceptual | Accountability was conceptually defined and separated into three distinct dimensions responsibility, answerability, and liability. | Provided framework for systematically analyzing accountability in administrative systems. | Perspectives and framework are theoretical in nature. |
| Kraak et al., 2014 | Global | Review | Developed a conceptual framework for assessing the accountability of food policies/environments including actors and processes involved. | Framework allows for systematically evaluating impacts on health and sustainability of policy/programmatic approaches. | Framework was not empirically applied or tested. |
| Šehović et al., 2018 | Global | Review | Explored conceptualizations and models of governance accountability, particularly pertaining to healthcare. Argued accountability is often fragmented and overlapping in practice. | Drew attention to 'accountability gaps' as an area requiring concerted analysis and action. | Perspectives presented are conceptual/theoretical in nature. |
| Mathai et al., 2015 | Global | Review | Examined accountability strategies that have been proposed or applied to end preventable maternal mortality. | Concluded multi-level, multi stakeholder accountability approaches with defined roles are needed to achieve health targets. | No primary research on effectiveness of proposed approaches was conducted. |
| Scott et al., 2016 | Global | Review | Assessed different accountability approaches for improving maternal and newborn health outcomes that have been suggested. | Determined various stakeholder groups must be engaged at multiple levels of governance. | Review nature limits conclusions able to be drawn about impact of different approaches. |
| Bandali et al., 2016 | Global | Review | Discussed the potential role of strengthened surveillance and response systems in supporting efforts to increase transparency and drive accountability. | Stressed importance of real time reviews, data sharing and corrective action planning as means to close audit loops. | Focus primarily on experience in sub-Saharan Africa and Asia. |
| Karlsson Vinkhuyzen et al., 2018 | Global | Review | Analyzed emerging conceptual frameworks and initiatives for strengthening accountability towards delivering the Sustainable Development Goals. | Highlighted need for mutual and cross sectoral SDG accountability that effectively integrates sustainability, policy coherence and governance. | Analysis is conceptual rather than based on empirical tests of frameworks. |
| Díaz Castro et al., 2022 | Global | Systematic Review | Reviewed analytical frameworks used in governance evaluations of health emergency policies, identifying commonly assessed domains and tools used. | Standardized evaluation approaches and analytical frameworks needed in health emergency policy governance assessments. | Included few Latin American studies. |
| Bennett et al., 2018 | Global | Review | Discussed challenges of multi sector governance for health due to differing mandates and capacities between sectors. | Governance arrangements tailored specifically to health issues and country level political, economic, and social contexts. | Perspectives presented are conceptual. |
| Jha et al., 2018 | Global | Systematic review | Synthesized evidence on risk communication strategies during health emergencies, finding varied practices across phases of emergency management. | Developed risk communication guidelines for integrating ERC as standardized practice across emergency phases globally. | Integrated few non English studies. |
| Hogan et al., 2008 | Global | Review | Assessed cross jurisdictional coordination challenges for public health law and policy in emergencies. | Establish coordinating bodies and plans for cross sectoral legal preparedness in health emergencies. | Did not assess implementation or impact of recommendations. |
| Finnigan, 2023 | Global | Review | Reviewed disaster policies in Asia Pacific nations, finding varied approaches. | Disaster policy frameworks integrating health across DM phases while ensuring continuity of care services during shocks. | Single researcher perspective presented. |
| Kamal et al., 2022 | Asia, Bangladesh | Qualitative | Applied disaster accountability framework to analyze Bangladesh system's strengths and gaps. | Mainstreaming disaster risk reduction into development planning processes and empowering local governance capacities. | Context specific to single country. |
| Gooding et al., 2022 | Global | Review | Synthesized lessons on emergency management partnership models. | Strengthening national and local partnerships and health emergency management systems through institutionalizing coordination models. | Countries studied varied. |
| Bascolo et al., 2022 | Global | Commentary | Discussed contributions of Public Health Functions Framework to COVID 19. | Use of new Public Health Functions Framework to strengthen global core capacities. | Perspectives conceptual. |
| Kassahun et al., 2017 | Africa, Ethiopia | Qualitative | Introduced accountability framework for polio eradication, finding improved performance monitoring and program implementation after 1 year. Identified strengths and gaps in the framework. | Expand implementation and evaluations to strengthen accountability framework approach for disease control programs. Refine framework based on ongoing monitoring to address gaps identified. | Results from single program implementation. |
| Otomo et al., 2014 | Global | Review | Summarized panel discussions on varied disaster preparedness frameworks, policies and challenges across countries/regions relating to healthcare management. | Establish guidance on core components for frameworks and policies on medical preparedness, disaster risk reduction and healthcare delivery. Harmonize approaches globally considering contextual factors. | Perspectives from panel discussion. |
| Surianto et al., 2019 | Global | Systematic review | Reviewed regional disaster risk reduction policies within Sendai Framework, finding wide variations in scope, definitions and components addressed. | Develop standardized minimum guidelines for essential elements to be included in regional DRR policies based on evidence from effective policies and programs. | Included few studies and regions. |
| Khan et al., 2018 | Global | Review | Proposed public health emergency preparedness conceptual framework focusing on individual and organizational resilience building strategies. | Pilots test the framework and refine indicators to empirically validate components. Develop tools and guidance for real world application across contexts. | Not empirically validated. |
| Craig et al., 2010 | Global | Commentary | Proposed policy framework focusing on core functions like surveillance, labs, logistics for emergency preparedness/response. | Standardize minimum functions and evaluate framework implementation considering resource constraints. Develop guidance toolkit for application tailored to country capacities/needs. | Not empirically validated. |
| Bazeyo et al., 2013 | Africa, Eastern | Review | Assessed institutional and coordination gaps for multi country epizootic emergencies in six Eastern African countries. | Propose standardized regional framework to strengthen coordination and roles of institutions involved. Address gaps in capacities, information systems and communication channels. | Regionally focused. |
| Mayega et al., 2013 | Africa, Uganda | Review | Analyzed gaps in disaster management policy, institutions, and roles responsibilities in Uganda. | Strengthen national policy framework based on good practices identified to address coordination structures and functions, resource allocation, roles at all levels. | Context specific to single country. |
| Era, 2021 | Asia, Philippines | Qualitative | Compared vertical and horizontal accountability strengths/limitations in Manila disaster management structures. | Integrate both vertical and horizontal accountability approaches tailored to context. Formalize multi-level coordination arrangements considering governance challenges. | Focused on two cities. |
| Osei & van Niekerk, 2007 | Americas, Jamaica | Qualitative | Analyzed policy and institutional network enablers and barriers for post disaster reconstruction in Jamaica. | Develop standardized guidelines for multi sector coordination frameworks and partnership models for disaster recovery. | Focused on single past disaster. |
| Jayasinghe et al., 2020 | Europe | Qualitative | Assessed lessons from 2010 11 earthquake recovery on governance, accountability, and participation. | Incorporate lessons on collaborative and adaptive management approaches. Institutionalize mechanisms for incorporating community and political participation. | Context specific to single past disaster. |
| Olu et al., 2016 | Africa | Review | Proposed integrated multi sectoral approaches for health disaster risk management in Africa. | Implement people centered programs and evaluate models of multi sector coordination for health considering variations in Africa. Develop standardized tools. | Recommendations were conceptual. |
| Tambo, 2017 | Africa | Review | Proposed improving DRR and emergency response coordination in African countries. | Conduct research on coordination models integrating health across phases. Develop standardized guidelines considering cross border, urbanization, and climate change factors in Africa. | Context specific to single region. |
| Ali et al., 2022 | Africa, Ethiopia | Qualitative | Explored multi sectoral COVID 19 response coordination approaches in Ethiopia. | Document models and establish coordination systems for future emergencies. Assess legal/regulatory enablers for multi-agency partnerships. | Focused on single country experience. |
| Neville et al., 2016 | Global | Review | Proposed development of DSS for cross border emergency multi agency decision making. | Develop and pilot test DSS prototype for evaluations. Integrate learning from usages to support adaptable standardized frameworks. | Perspectives were conceptual. |
| Van Niekerk, 2014 | Africa, South Africa | Review | Analyzed SA disaster management policy and legislative gaps. | Revise frameworks based on internationally benchmarks best practices. Strengthen coordination, roles, and resources allocation across tiers. | Context specific to single country. |
| Mwendera, C., et al. 2017 | Africa, Malawi | Qualitative study involving stakeholder interviews and workshops | The study developed a framework for improved utilization of malaria research for policy development in Malawi. Key elements of the framework included enhancing the dissemination of malaria research findings to relevant policymakers and stakeholders, facilitating active engagement between researchers and policymakers, and supporting evidence informed policy development processes. | The researchers recommended implementing the framework to strengthen the linkage between malaria research and policy in Malawi. This would help ensure that research evidence is better communicated to relevant audiences and actively considered in policymaking processes. | The study had a small sample size, and its findings may not be generalizable to other countries. The framework was also not empirically tested. |
| Zhang, H.J.J.o.C.P.A.R. and Practice 2012 | Asia, China | Literature review and content analysis | The study found that China has improved its emergency management system after major disasters like SARS, snowstorms, and earthquakes through establishing new emergency response agencies at various government levels, strengthening coordination mechanisms across agencies, improving early warning systems, and increasing investment in hazards mitigation. | The study recommended that China should further improve multi-level and inter agency coordination mechanisms to strengthen collaboration across different stakeholders involved in emergency response. It also suggested increasing community and public engagement in preparedness and response activities. | The study only focused its analysis on three major disasters in China and may have missed other important factors influencing changes in China's emergency management system over time. |
| Panneer, S., et al. 2024 | Global | Literature review | The study found that multistakeholder participation, collaboration and networking across different organizations and sectors are key factors for effective disaster risk reduction and pandemic management. It improves coordination, information sharing, joint planning, and efficient mobilization of resources. | The study recommended institutionalizing multisectoral coordination structures and partnerships at all levels of governance to strengthen collaborative emergency preparedness and response. | The study was based on a review of existing literature rather than primary empirical research. It did not examine real examples of multistakeholder collaboration on the ground. |
| Bowen, T., et al.2020 | Global | Literature review | The study found that establishing institutional arrangements like permanent coordination bodies and forming partnerships between humanitarian, development and other sectors are critical for multisectoral collaboration, coordination of efforts and linkages between humanitarian assistance and long-term development actions. | The researchers recommended that such institutional arrangements and partnerships should be established and strengthened to facilitate improved coordination among diverse sets of actors involved in emergency response and recovery efforts. | The study was based on a review of literature and did not involve primary empirical research. It was also a broad overview without focus on specific case studies. |
| Sawanpanyalert, P. 2006 | Asia | Literature review | The study found that establishing regional and global networks of collaboration among countries through sharing expertise, training, equipment, and joint research improves capacities for disease surveillance, detection, research, and management of emerging and re-emerging infectious diseases across borders. | Based on the findings, the study recommended that authorities should aim to strengthen existing regional and global collaboration networks and partnerships to bolster infectious disease prevention, control, and health security. | As a literature review, the study did not involve primary empirical research. It also focused only on disease control outcomes without examining other dimensions of multi country cooperation. |
| Olson, R.S. 2018 | Global | Conceptual paper | The study argued that key elements like public accountability and transparency in use of resources, engagement and consultation of affected communities and stakeholders, and visible political leadership and commitment are crucial factors for motivating action and investments in disaster risk reduction. | The author recommended that strategies and mechanisms should be implemented to ensure public accountability of relevant agencies, transparent communication with people, and methods to induce interest and will for DRR among political leadership and policymakers. | The paper involved a conceptual framework without any empirical analysis or testing of the hypotheses regarding factors influencing DRR efforts. |
| Camargo, C.B. and E.J.B.I.o.G.W.P. Jacobs 2011 | Global | Conceptual paper | The paper proposed a conceptual framework and indicators for assessing key dimensions of health system governance like policy formulation, resource management, community participation, as well as regulation and corruption monitoring. The framework aimed to measure stewardship qualities in low-income national health systems. | The researchers suggested the assessment framework can help prioritize actions and guide strategic investments for strengthening governance functions to boost health system performance and outcomes. However, the framework was not empirically tested and would need to be contextualized for practical use in specific country settings and health system models. | The framework was not empirically tested and would need to be contextualized for practical use in specific country settings and health system models. |
| Fitzgerald, M.R., et al. 2023 | North America | Retrospective study | The study found that adopting a multi-agency coordination framework involving public health and emergency management agencies strengthened collaboration between agencies through joint planning, resource sharing, training, and collaborative operations during the COVID19 response in a healthcare setting. | Researchers recommended institutionalizing coordination mechanisms through formal agreements, regular trainings, and exercises to maintain partnerships and improve multi agency collaboration for future public health emergencies. | The single system case study design limits generalizability. Comparison with other organizational models or broader contexts was also not perform |
| Tong, X., et al. 2020 | Asia, China | Case study | The study identified specific capacities and indicators to assess local governments' emergency response capability in Shenzhen, China. The developed framework provided structure and measures for evaluation. | The researchers recommended applying and testing the assessment framework in other local government settings to validate it. They also suggested future studies examine multi-level governance dynamics influencing emergency response capacity. | The case study was limited to one local context. |
| Suryaman, Y. 2020 | Asia, Indonesia | Case study | The study identified several accountability issues through interviews and document review of the flooding response in Garut, West Java, Indonesia including coordination gaps and limited community participation. | The researcher recommended strengthening coordination between disaster response agencies through clear standard operating procedures. Enhancing community participation and feedback mechanisms in disaster response planning and implementation was also advised. | The study examined one past event in a localized context. |
| Handmer, J. and S. Dovers 2013 | Global | Handbook | The handbook discussed various disaster policies, institutions, models, and frameworks from international experience based on existing literature. | Recommendations focused on further research and practice to better align policies and coordinate multi-level, multi sector responses globally. The handbook also emphasized utilizing adaptive governance principles. | The analysis relied on secondary sources rather than primary data. |
| Jacobson, P.D., et al. 2012 | Global | Conceptual analysis | The study discussed conceptual opportunities and challenges around the role of law in public health preparedness based on a literature review. | The authors called for further multidisciplinary research at the interface of law and public health preparedness to strengthen policy and practice. | The analysis relied on existing knowledge and did not involve primary research. |
| Villumstad, S. and B.J.D. Hendrie 1993 | Africa, Ethiopia | Policy analysis | The paper described achievements and ongoing deficiencies in Ethiopia’s disaster preparedness and response based on a review of policies and documents. | Recommendations focused on enhancing community resilience, coordination mechanisms, hazard monitoring and preparedness planning approaches. Long term capacity building was also emphasized. | The analysis was based on existing policies and documents rather than new empirical work. |
| Aliyu, A.J.A.o.A.M. 2015 | Africa | Literature review | The paper reviewed literature to analyze challenges in disaster/emergency management in Africa and identified gaps in coordination, preparedness, and response. | Recommendations included strengthening multi sector collaboration, community engagement, disaster risk reduction frameworks and investment in early warning systems. | Secondary sources and existing knowledge were drawing upon rather than primary data collection. |
| Di Giulio, G.M., et al.,2023 | South America, Brazil | Qualitative analysis of governance during COVID 19 | The study identifies significant gaps in Brazil's emergency governance, highlighting delays in decision making and poor communication among agencies. It emphasizes the chaos caused by fragmented responses and the lack of a cohesive national strategy. | Enhance governance frameworks by establishing clear communication channels and protocols among agencies. Develop a national emergency response plan that integrates local needs and leverages community engagement. | Limited to Brazil; findings may not generalize to other countries with different governance structures. |
| Shah, I., et al.,2020 | Asia, Pakistan | Case study on institutional arrangements | The research reveals that weak institutional frameworks hinder effective disaster risk management in Pakistan. Key issues include inadequate resource allocation, lack of coordination among agencies, and insufficient training for personnel. | Strengthen institutional frameworks by enhancing inter agency collaboration and increasing funding for disaster preparedness programs. Implement regular training and capacity building exercises for relevant stakeholders. | Focus on specific institutions; limited scope may overlook broader systemic issues affecting disaster management. |
| Shahbaz, S.J.P. et al, 2019 | Asia, Pakistan | Systematic review | The review synthesizes findings from various studies, indicating that Pakistan's disaster management system is reactive rather than proactive. It highlights challenges such as limited community involvement and outdated policies that fail to address current risks. | Improve coordination among agencies by developing integrated disaster management policies that involve community input. Promote public awareness campaigns to enhance community preparedness and resilience. | Limited to existing literature; may miss emerging issues and innovations in disaster management practices. |
| Nawab, B. et al, 2017 | Asia, Various | Analytical literature review | The analysis reveals that climate change exacerbates existing vulnerabilities in disaster management. Institutional complexities and lack of clarity in roles hinder effective responses to climate related disasters. | Prioritize integrated approaches that align disaster management with climate adaptation strategies. Foster collaboration among governmental and non-governmental organizations to streamline efforts. | Broad scope: lacks specific case studies that illustrate successful interventions or collaborative models. |
| Ainuddin, S. et al, 2012 | Asia, Pakistan | National to local level analysis | Findings indicate a disconnect between national policies and local implementation, resulting in ineffective disaster response mechanisms. Local governments often lack the authority and resources to act effectively in emergencies. | Foster local governance and community engagement in preparedness by decentralizing decision making processes. Ensure that local authorities have the necessary resources and training to implement national policies effectively. | Limited time frame lacks longitudinal data to assess the effectiveness of recommendations over time. |
| Cheema, A.R., et al.2016 | Asia, Pakistan | Policy analysis of disaster management | The study identifies structural weaknesses in Pakistan’s disaster management policies, including inadequate disaster risk assessments and insufficient integration of disaster response into development planning. | Recommend reforms based on historical lessons, including the establishment of a national disaster management agency with clear mandates and the integration of disaster risk reduction into development policies. | Focuses on past events; may not reflect current dynamics or the impact of recent disasters on policy changes. |
| Tahir, M.A., et al.2023 | Asia, Pakistan | Case study of operational strategies | The evaluation reveals that coordination challenges during the COVID 19 response led to inefficiencies and delays. Lack of a unified command structure complicated decision making and resource allocation. | Develop standardized protocols for emergency operations that ensure clear lines of authority and accountability. Conduct regular drills and simulations to test coordination mechanisms among various agencies. | Limited to COVID 19 context; findings may not apply to other types of disasters or emergencies. |
| Abdeen, F., et al. 2021 | Asia, Sri Lanka | Case study on multi agency collaboration | The study uncovers significant barriers to effective collaboration among agencies involved in disaster management in Sri Lanka. Issues include overlapping mandates, poor communication, and a lack of trust among organizations. | Enhance multi agency communication and coordination by establishing a centralized information sharing platform. Foster a culture of collaboration through joint training and exercises among different stakeholders. | Focus on specific case; may not generalize to all contexts within Sri Lanka or other countries. |
| Koswatte, I. et al, 2022 | Asia, Sri Lanka | Policy analysis | The analysis highlights gaps in Sri Lanka's crisis management policies, particularly in their responsiveness to emerging threats and integration of new risk assessments. It emphasizes the need for policies that are adaptable and forward looking. | Recommend comprehensive policy frameworks that are flexible and adaptable to changing circumstances. Encourage stakeholder engagement in policy development to ensure relevance and effectiveness. | Limited to policy level; lacks empirical data on the actual implementation of recommendations. |
| Rathnayake, W., et al.2021 | Asia, Sri Lanka, Myanmar, Maldives | Comparative case study | The investigation reveals that while some institutions have been established for disaster governance, there are gaps in empowerment and effectiveness. Case studies indicate that local capacities remain underutilized in all three countries. | Foster regional cooperation for disaster risk governance by sharing best practices and resources among countries. Strengthen local capacities by providing training and resources tailored to community needs. | Varied contexts may complicate comparisons; findings specific to each country may not be applicable to others. |
| Furukawa, S.i.J.J.o.C. et al, 2000 | Asia, Japan | Framework analysis | The study outlines a comprehensive framework for crisis management in Japan, emphasizing the importance of coordination among various sectors and the role of community involvement in enhancing resilience. | Recommend continuous updates to crisis management frameworks to incorporate new research findings and technological advancements. Engage communities in planning and response efforts to ensure relevance. | May be outdated; the framework may not reflect recent developments or lessons learned from new disasters. |
| Takao, Y.J.J.S.2023 | Asia, Japan | Analytical review | The review assesses the effectiveness of reforms implemented after the Fukushima disaster, noting improvements in accountability and community engagement but also highlighting ongoing challenges in disaster preparedness and response systems. | Strengthen shared responsibility and collaborative accountability by promoting transparency in decision making processes. Implement mechanisms for community feedback to enhance trust and efficacy in disaster response efforts. | Focuses on specific reforms; may overlook broader systemic issues affecting disaster management in Japan. |
| Ishiwatari, M.J.N.H.R.2021 | Asia, Japan | Case study of institutional coordination | The study emphasizes the critical role of engaging both national and local governments in disaster management. It identifies effective coordination as key to enhancing response capabilities and ensuring community needs are met during crises. | Enhance coordination mechanisms among government levels by establishing clear roles and responsibilities. Promote regular communication and joint training exercises to build trust and improve collaborative efforts. | Limited to Japan; findings may not be applicable to countries with different governance structures. |
| Okada, A., et al 2014 | Asia, Japan | Descriptive analysis | The analysis provides insights into recent developments in Japan's disaster management system, particularly in information flow and command structures, noting improvements but also areas needing further refinement. | Recommend improved information flow and command chains to facilitate quicker decision-making during emergencies. Regularly review and update communication protocols to adapt to new challenges. | Limited focus on specific developments; broader context needed to assess overall effectiveness. |
| Kanbara, S. 2021 | Asia, Japan | Policy analysis | The study analyzes Japan's disaster risk reduction regime, emphasizing the importance of open data and governance in improving disaster preparedness and response capabilities. | Promote open data and transparency in governance to enhance public trust and participation in disaster management. Encourage collaborative approaches that include multiple stakeholders in planning and response. | Focuses solely on governance aspects; may lack practical insights into on the ground implementation. |
| Kim, K.D. 2013 | Asia, Various | Network analysis | The research discusses the effectiveness of policy networks in coordinating disaster response efforts. It highlights the importance of collaboration across sectors to achieve comprehensive and timely responses to emergencies. | Encourage collaborative frameworks among stakeholders to enhance coordination and resource sharing during disasters. Develop training programs that focus on inter agency collaboration and communication. | Limited by the diversity of contexts; may lack specificity regarding successful models or practices. |
