# Supplementary material for: Governance, coordination, and accountability in public health emergency preparedness and response: A scoping review
Source: PLOS Glob Public Health. 2026 Sep 18;6(9):e0006719. doi: 10.1371/journal.pgph.0006719 (PMC13600703; doi:10.1371/journal.pgph.0006719)
Supplement: Supplementary file 7 [file pgph.0006719.s006.docx]

**Reviewer Report**

Manuscript Title: Governance, Coordination, and Accountability in Public Health Emergency Preparedness and Response: A Scoping Review

**Recommendation: Minor Revision**

**Summary of the Manuscript**

This manuscript presents a scoping review of 77 studies (2000–2025) examining governance, coordination, and accountability in public health emergency preparedness and response. Using PRISMA-ScR methodology, the authors identify key themes related to institutional arrangements, coordination mechanisms, and systemic challenges, with emphasis on multisector collaboration and policy frameworks.

The topic is highly relevant, particularly for low- and middle-income countries, and the manuscript compiles a substantial body of literature. However, important methodological, analytical, and interpretive limitations weaken its contribution in its current form.

Some Comments

1. Clarity and Strength of Main Claims

The manuscript argues that:

• Governance, coordination, and accountability systems are central determinants of preparedness.

• Fragmentation, weak accountability, and unclear mandates undermine emergency response.

• Strong systems correlate with legal clarity, coordination bodies, and performance monitoring.

Concerns

• The claims are broad and largely confirm existing consensus, rather than offering new insights.

• There is no clear differentiation between descriptive mapping and causal inference.

• The conclusion suggests influence (“preparedness is shaped by…”) without analytical justification beyond thematic synthesis.

Recommendation

• Reframe conclusions to reflect descriptive scope rather than causal interpretation, OR

• Strengthen analytical framing to justify causal language.

2. Engagement with Existing Literature

Strengths

• Wide coverage of global and regional literature

• Inclusion of both peer-reviewed and grey literature

• Relevant references to international frameworks (e.g., IHR)

Limitations

• Literature is summarized rather than critically synthesized

• Limited engagement with:

Competing governance theories

Differences across political or health system contexts

• Minimal discussion of contradictory evidence or debates

Recommendation

• Introduce critical comparison across studies

• Highlight where evidence diverges or is inconsistent

• Clarify what this review adds beyond prior reviews

3. Methodological Rigor and Transparency

Strengths

• PRISMA-ScR framework followed ✔️

• Clear eligibility criteria ✔️

• Dual screening with inter-rater reliability (kappa = 0.82) ✔️

Limitations

• No formal quality appraisal (acceptable for scoping, but limits interpretation)

• Insufficient detail on:

Data extraction framework (not fully presented)

Coding process

Thematic development

Critical Issue

The manuscript explicitly states:

Better to clearly state causal or effectiveness assessment was conducted

Yet conclusions imply:

• Relative importance of governance factors

• Policy effectiveness

Recommendation

• Clarify analytical boundaries

• Provide supplementary materials:

Data extraction template

Coding framework

Example coded data

4. Data and Evidence Supporting Claims

Findings

• Themes are clearly presented and logically structured.

• Evidence is heterogeneous (conceptual, empirical, policy reports).

Concerns

• Provide cleared distinction between:

Strong vs weak evidence

Empirical vs conceptual studies

• No weighting or hierarchy of evidence

Implication

• Conclusions risk overgeneralization.

Recommendation

• Clearly distinguish:

Types of evidence

Strength of support

• Avoid language implying effectiveness or causality

5. Use of Grey Literature

Strength

• Inclusion adds policy relevance

Concern

• Grey literature is treated equally to peer-reviewed studies

• No discussion of:

Potential bias

Differences in methodological rigor

Recommendation

• Explicitly acknowledge limitations of grey literature

• Consider separating synthesis or interpretation

6. Need for Conceptual or Analytical Framework

Currently, the manuscript:

• Identifies themes

• Describes relationships

But does not

• Integrate findings into a unified framework

Recommendation

Develop a:

• Conceptual model of governance-accountability-preparedness

• Or analytical framework showing relationships between:

Governance structures

Coordination mechanisms

Outcomes

This would significantly strengthen contribution.

7. Reproducibility and Data Availability

Current Status

• Authors state all data are publicly available

Limitations

• No publicly available dataset of:

Included studies

Extracted variables

• Search strategy not fully reproducible within manuscript

Recommendation

• Provide:

Full search strings

Extraction dataset (as supplementary file)

8. Reporting Guidelines Compliance

• PRISMA-ScR: largely followed ✔️

• Arksey & O’Malley framework: applied ✔️

Minor Gaps

• No protocol registration

• Limited reporting of:

Data charting process

Analytical decision-making

9. Writing, Structure, and Accessibility

Strengths

• Logical structure

• Clear sectioning

• Accessible language for multidisciplinary audience

Weaknesses

• Repetition of key terms without added analytical depth

• Some sections overly descriptive

• Discussion occasionally reiterates results rather than interpreting them

Recommendation

• Reduce redundancy

• Strengthen analytical interpretation in Discussion

Minor Comments

Ensure consistent terminology (e.g., “multi sector” vs “multisector”)

Clarify time frame inconsistencies (some sections report 2000–2020 vs 2000–2025)

Improve clarity of figures/tables (not fully assessable in text extract)

Remove minor typographical inconsistencies

**Responses to Specific PLOS Criteria**

**Criterion Assessment**

Main claims & significance: Major contribution; needs some analytical positioning

Placement in literature: Adequate

Data support claims: Partially supported; risk of over interpretation

Protocol adherence: Good adherence

Need for protocols/algorithms: Yes – conceptual framework recommended

Suitability for publication: Yes, after minor revision

Data availability: Acceptable

Guideline compliance: Generally compliant

Method reproducibility: Moderate; needs more transparency

Software availability: Not applicable

Clarity and accessibility: Good, but repetitive

Final Recommendation

**Minor Revision Required**

Key Actions for Authors (Priority Order)

1. Align conclusions with methodology: Avoid causal language unless justified

2. Add conceptual/analytical framework: Move beyond descriptive synthesis

3. Improve methodological transparency: Provide extraction and coding details

4. Differentiate evidence types: Avoid treating all sources equally

5. Strengthen discussion: Interpret, not just restate findings

**Closing Assessment**

This manuscript addresses very important and policy-relevant topic, and the dataset is valuable. If some improvements made, it helps for policy makers and its contribution to the field is vital.

With substantial revision—particularly strengthening analytical depth and methodological clarity—the paper could reach the standard expected for publication.
