# Supplementary material for: Governance, coordination, and accountability in public health emergency preparedness and response: A scoping review
Source: PLOS Glob Public Health. 2026 Sep 18;6(9):e0006719. doi: 10.1371/journal.pgph.0006719 (PMC13600703; doi:10.1371/journal.pgph.0006719)
Supplement: Supplementary file 8 [file pgph.0006719.s009.docx]

**RESPONSE TO REVIEWERS**

**Governance, Coordination, and Accountability in Public Health Emergency Preparedness and Response: A Scoping Review**

**Manuscript:** PGPH-D-26-00735 **Journal:** PLOS Global Public Health

**Corresponding author:** Sileshi Demelash Sasie, Ethiopian Public Health Institute (sileyeshi21@gmail.com; ORCID 0000-0002-7273-8923)

Dear Dr. Catherine Grant and Reviewers,

We thank the Academic Editor and all three reviewers for their careful, constructive, and generous appraisal of our manuscript. We are encouraged that the reviewers found the topic timely and policy-relevant, the methods clearly described, and the contribution meaningful for both national and global audiences. We are grateful for the recommendation of minor revision.

We have addressed every point raised. The most substantive revisions concern (i) the systematic removal of causal and evaluative language throughout the abstract, results, discussion, policy implications, and conclusion, replacing it with descriptive, mapping-consistent phrasing (“associated with,” “reported alongside,” “identified in relation to”); (ii) the merging of former Themes 1 and 5 to eliminate conceptual overlap; (iii) explicit differentiation of evidence types (empirical, conceptual, grey literature) within the synthesis and tables; (iv) the addition of a conceptual framework figure integrating governance, coordination, and accountability; and (v) enhanced methodological transparency through three new supplementary files (data-extraction template, coding framework, and full search strings).

Below we respond to each comment in turn using a Comment – Response – Revision structure. For transparency, all changes are shown in **green** in the tracked-changes manuscript, and revision text quoted in this letter is likewise rendered in green. Page and line references correspond to the revised manuscript unless otherwise stated.

**Part A Editor’s comments (required for acceptance)**

**A1. Analytical framing and conclusions (causal/evaluative language; Results–Discussion redundancy)**

| **COMMENT** | *“Conclusions at times imply causal or evaluative claims (e.g., that certain governance factors ‘shape’ or determine preparedness outcomes) that are not analytically justified within a scoping review reframe conclusions using language such as ‘associated with,’ ‘reported alongside,’ or ‘identified in relation to’ The discussion should interpret findings rather than restate results.”* |
| --- | --- |
| **RESPONSE** | We fully agree and have taken option (a). We undertook a systematic, line-by-line audit of the manuscript for causal and evaluative constructions and replaced them with mapping-consistent language throughout. This included the verbs *shape / shaped by / determine / drives / undermine / most strongly shaped* and the evaluative phrasing *“strongest where”*, which we have reframed as associations or as patterns reported in the literature.  We have additionally chosen our words to reflect that the unit of evidence is the reporting study, not the emergency system itself; the review maps what the literature reports, and the revised text now says so explicitly at the points the reviewers identified (former lines 18, 24, 42, 263, 269, 271, 281, 285).  To reduce Results–Discussion redundancy, we restructured the Discussion so that each paragraph now opens with an interpretive proposition (what the pattern means, where evidence diverges, and what this review adds beyond prior reviews) rather than re-summarizing thematic content. Approximately 320 words of restated results were removed and replaced with interpretation and critical comparison. |
| **REVISION** | Abstract – Conclusions: “This review maps how effective public health emergency preparedness is described in the literature less in relation to resources alone than to the strength and integration of governance, coordination, and accountability systems. Stronger preparedness was reported alongside adaptive governance, institutionalized multisectoral coordination, transparent oversight, and continuous learning.”  Conclusion (¶1): “multisector accountability is described across the literature in relation to how governance frameworks, legal authority, institutional mandates, coordination structures, and political–administrative conditions interact recurring challenges were consistently reported alongside weaker preparedness and response as described in the included studies.”  Policy Implications (opening): “Across the evidence, multisector emergency preparedness was most often reported in relation to whether legal authority, institutional mandates, and accountability mechanisms were enforceable in practice, rather than the mere presence of policies” (Full set of edits visible in green in the tracked manuscript.) |

**A2. Methodological transparency (extraction template, codebook, search strings)**

| **COMMENT** | *“Provide, as supplementary materials: the full data extraction template, the coding framework or codebook, and the complete search strings used essential for reproducibility.”* |
| --- | --- |
| **RESPONSE** | We agree this strengthens reproducibility and have prepared three supplementary files, now explicitly cited in the Methods: S3 File (data-extraction template, with all variables and definitions), S4 File (the complete coding framework / codebook, including the six-phase thematic process, all codes and definitions, candidate-to-final theme aggregation, and analytical decisions), and S2 File (the full, database-specific Boolean search strings for all five databases with retrieval counts and the last-search date).  The codebook reproduces the exact code set applied during analysis (e.g., LEGAL_FRAMEWORK, ROLE_AMBIGUITY, CENTRALIZED, DECENTRALIZED) and shows how codes were aggregated into candidate and then final themes, so that the analytical chain from raw text to theme is fully auditable. |
| **REVISION** | Methods – Data extraction: added “The complete data-extraction template, including all charted variables and their definitions, is provided in S2 File.”  Methods – Data analysis: added “The full coding framework comprising all codes, definitions, the six-phase analytical process, candidate-to-final theme mapping, and cross-cutting analytical decisions is provided in S3 File to support transparency and reproducibility.”  Methods – Search: added “The full electronic search strategy for all five databases, including exact Boolean strings, field tags, limits, and records retrieved per database, is presented in S1 File.” |

**A3. Evidence differentiation (empirical vs conceptual vs grey literature)**

| **COMMENT** | *“The manuscript treats conceptual papers, empirical studies, and grey literature as equivalent clearly differentiate evidence types across themes indicate whether claims are supported by empirical data, conceptual argument, or policy documentation and explicitly acknowledge the limitations of including grey literature.”* |
| --- | --- |
| **RESPONSE** | We have addressed this in three ways. First, we added an evidence-type classification (empirical / conceptual-theoretical / review-framework / grey-literature policy) to the study-characteristics description and to the S4 study-characteristics table, so each source’s evidence type is explicit. Second, within each theme we now signal the evidence base of key statements (e.g., “empirical event-based studies described,” “conceptual analyses proposed,” “grey-literature evaluations reported”), so readers can see the type of evidence behind each pattern. Third, we kept the grey-literature synthesis (Table 3) visibly separate from the peer-reviewed synthesis and added an explicit limitation.  We also note in the text that, consistent with scoping-review methodology, evidence types are differentiated descriptively rather than ranked in a formal hierarchy of quality we make this analytical boundary explicit so that differentiation is not mistaken for graded appraisal. |
| **REVISION** | Results – Characteristics: added “Included sources comprised empirical studies (n= ), conceptual or theoretical analyses (n= ), reviews and framework analyses (n= ), and grey-literature policy and evaluation documents (n=10); evidence type is indicated for each source in S4 Table.”  Limitations: added “Grey-literature sources were included for their policy relevance but are not peer-reviewed and vary in methodological transparency; they were therefore synthesised and presented separately (Table 3) and interpreted with appropriate caution, and were not weighted equally with peer-reviewed evidence in the synthesis.” |

**A4. Thematic clarity (overlap between Theme 5 and Theme 1)**

| **COMMENT** | *“Theme 5 (centralization and decentralization) overlaps substantially with Theme 1 (governance architecture). Either clarify the analytical distinction or merge them with appropriate restructuring.” (Editor; Reviewer 2, point 3)* |
| --- | --- |
| **RESPONSE** | We agree and have merged former Theme 5 into Theme 1. The review now presents four higher-order themes rather than five. The centralization–decentralization material is now integrated as a dedicated sub-section within Theme 1 (“Governance architecture, legal foundations, and the distribution of authority”), because the centralization–decentralization continuum is analytically a property of governance architecture rather than a separate theme. No evidence was lost in the merge; all sources previously under Theme 5 are retained within the expanded Theme 1, and the coding framework (S4 Table) reflects this consolidation. |
| **REVISION** | Results – theme list now reads: “identified four higher-order analytical themes (i) governance architecture, legal foundations, and the distribution of authority (including centralized and decentralized arrangements); (ii) institutional roles and coordination mechanisms; (iii) accountability failures and system fragmentation; and (iv) reform approaches and enabling conditions.” The former Theme 5 paragraph is relocated under Theme 1 as a subsection; theme numbering updated throughout; S3 File codebook updated. |

**Part B Editor’s recommendations (strengthening the manuscript)**

**B1. Conceptual / analytical framework figure**

| **COMMENT** | *“Develop a conceptual model or diagram that integrates governance structures, coordination mechanisms, and accountability into a unified framework a figure on page 5 (line 102) would substantially improve the manuscript’s contribution and accessibility.” (Editor; Reviewer 3 point 6; Reviewer 1)* |
| --- | --- |
| **RESPONSE** | We welcomed this suggestion and have developed a conceptual framework (new Figure 2) that integrates the three constructs into a single descriptive model. The framework depicts governance architecture and legal foundations as the enabling layer; institutional roles and coordination mechanisms as the operational layer; accountability systems (oversight, monitoring, feedback, community accountability) as the connective layer that binds the other two; and reform/enabling conditions and the centralization–decentralization continuum as cross-cutting modifiers. Arrows denote reported relationships rather than demonstrated causal effects, consistent with the descriptive scope of the review. |
| **REVISION** | Added new Figure 2 (conceptual framework) and a callout in the Methods/Results bridging text: “The relationships among governance, coordination, and accountability constructs mapped in this review are summarised descriptively in Figure 2.” Figure provided separately in .tif/.eps per journal requirements. |

**B2. Country/regional examples per theme; Ethiopia balance**

| **COMMENT** | *Reviewer 1: “Provide an example for each theme a tangible example from a certain country context. Otherwise, it feels too amorphous.” Reviewer 2: “Streamline the Ethiopia section to avoid imbalance with the global discussion.”* |
| --- | --- |
| **RESPONSE** | We have added a concise, concrete country example to each theme to anchor the synthesis, drawn from sources already in the evidence base: e.g., Theme 1 post-Hurricane Katrina (USA) role-clarity gaps and post-Ebola legislative reform (Guinea); Theme 2 vertical/horizontal coordination in the Philippines (Manila) and EOC structures; Theme 3 fragmentation in Bangladesh’s disaster-accountability analysis; Theme 4 nutrition/community feedback mechanisms in Burkina Faso and the Sendai-aligned reforms. To preserve balance (Reviewer 2), we simultaneously condensed the Ethiopia material in the Background by approximately 40%, retaining it as one illustrative example rather than an extended national case. |
| **REVISION** | Each theme now contains one italicized exemplar sentence, e.g., Theme 1: “For example, post-Hurricane Katrina analyses in the United States described how ambiguous role definition was reported alongside coordination breakdowns, while post-Ebola legislative reforms in Guinea were described as strengthening accountability mandates**.**” Background Ethiopia paragraphs condensed (green strike/insert in tracked file). |

**B3. Table 4 factor examples**

| **COMMENT** | *Reviewer 1: “Table 2 it would be helpful to have examples of each of the factors to explain what this means practically within a national context.”* |
| --- | --- |
| **RESPONSE** | We added a short **“**Illustrative example**”** column to Table 2 giving a practical, country-anchored instance for each factor (e.g., for “Legal mandates to enforce compliance”: IHR (2005) core-capacity obligations enacted in national public-health law; for “multi-sectoral coordination bodies”: national EOCs/incident-management committees). This keeps the descriptive-mapping purpose while making each factor concrete. |
| **REVISION** | Table 4 expanded with an “Illustrative example (as reported. |

**Part C Reviewer 1: specific queries**

| **COMMENT** | *Page 3, line 66: “Many countries have yet could they specify what percentage and where? Which countries?”* |
| --- | --- |
| **RESPONSE** | We have added quantification from the WHO IHR State Party Self-Assessment Annual Reporting (SPAR) and Joint External Evaluation evidence base. We specify that, although nearly all WHO Member States report legislative alignment, a substantial share continue to report limited implementation of enforceable legal and financing mechanisms, and we name illustrative regions/countries reported in the included literature (e.g., settings in sub-Saharan Africa and South Asia such as Uganda, Bangladesh, and Pakistan). |
| **REVISION** | Background: “available evidence indicates that many countries including a substantial proportion of low- and middle-income States Parties reporting weaker IHR legal and financing capacities in SPAR/JEE assessments (for example, settings such as Uganda, Bangladesh, and Pakistan described in the included literature) have yet to fully implement the required legislative and administrative mechanisms**.**” |

| **COMMENT** | *Page 14, line 270: the decline in publications 2021–2024 despite COVID-19 any explanation?* |
| --- | --- |
| **RESPONSE** | This is an astute observation. The apparent decline is most plausibly an artefact of indexing and publication lag rather than a true reduction in scholarship: (i) database indexing lag means 2023–2024 outputs were incompletely captured at the search date (05 June 2025); (ii) our eligibility window and English-language restriction excluded a portion of pandemic-era output; and (iii) much COVID-19 governance writing appeared as commentaries/editorials outside our governance-and-accountability inclusion criteria. We now state this explicitly and avoid interpreting the trend substantively. |
| **REVISION** | Results – Characteristics: added “The comparatively smaller number of sources indexed for 2021–2024 likely reflects database indexing and publication lag at the search date, the English-language restriction, and the exclusion of non-governance commentary, rather than a genuine decline in pandemic-era scholarship; this temporal pattern should therefore be interpreted with caution.” |

| **COMMENT** | *Page 20, Table 3: “I didn’t understand this table. What is it trying to say and why were these publications pulled out?”* |
| --- | --- |
| **RESPONSE** | We apologies for the lack of clarity. Table 3 presents the grey-literature sources only (n=10) deliberately separated from peer-reviewed studies to show, transparently, what governance/accountability features and what implementation gaps each policy or institutional document described. We have rewritten the title, added an explanatory lead-in sentence, and clarified the column logic so the table is self-explanatory. Retaining it (rather than dropping it) directly supports the evidence-differentiation point (A3) by making the grey-literature contribution visible and auditable. |
| **REVISION** | New Table 3 title: “Grey-literature sources (n=10): governance and accountability features described and implementation gaps reported, presented separately from peer-reviewed evidence**.**” Added lead-in: “Table 3 summarises the ten grey-literature documents only; these are reported separately from peer-reviewed studies to make the type and source of evidence explicit**.**” |

| **COMMENT** | *Page 23, line 434: “Are the authors saying that political leadership and resources like money don’t matter? I wasn’t clear what this line is saying.”* |
| --- | --- |
| **RESPONSE** | We did not intend that reading and have rewritten the sentence. Our point is the opposite: leadership and financing matter greatly, but the literature reports that they operate through governance design that is, the same resources and political commitment are reported to yield stronger preparedness when authority, mandates, and accountability are clearly configured, and weaker preparedness when they are fragmented. The clause now states this explicitly. |
| **REVISION** | Discussion: “This does not imply that political leadership or financing are unimportant; on the contrary, the literature describes them as decisive enabling conditions. Rather, the evidence suggests that leadership and resources are reported to translate into preparedness most consistently when they are channeled through clear authority, defined mandates, and functioning accountability mechanisms, and least consistently where these governance arrangements are fragmented.” |

| **COMMENT** | *Page 27, line 531: enforceability of policies “do they have any thoughts or examples of how to do this?”* |
| --- | --- |
| **RESPONSE** | We have added concrete, literature-grounded mechanisms for moving from policy adoption to enforceable practice: statutory assignment of a lead coordinating authority with budgetary control; binding inter-agency memoranda with defined performance obligations; linking Joint External Evaluation / after-action-review findings to mandated, time-bound corrective actions; and independent oversight with published compliance reporting. Examples (e.g., post-Ebola legislative tools in Guinea; polio-eradication accountability frameworks) are cited from the included evidence. |
| **REVISION** | Policy Implications: added “Enforceability may be strengthened through mechanisms reported in the literature, such as statutory designation of a lead authority with budget control, binding inter-agency agreements specifying performance obligations, and the linkage of JEE and after-action-review findings to mandated, time-bound corrective actions with independent, publicly reported oversight (for example, the post-Ebola legislative assessment tool in Guinea and accountability frameworks applied in polio eradication).” |

**Part D Reviewers 2 and 3: additional points**

| **COMMENT** | *Reviewer 2: “Clarify whether the search strategy itself was independently verified by a second reviewer.”* |
| --- | --- |
| **RESPONSE** | We have clarified that the search strategy was peer-reviewed by a second senior reviewer prior to execution, in addition to the dual independent screening already described. |
| **REVISION** | Methods – Search: added “The search strategy was independently reviewed for completeness and accuracy by a second reviewer before execution, consistent with PRESS-style peer review.” |

| **COMMENT** | *Reviewer 3: “Literature is summarized rather than critically synthesized limited engagement with competing governance theories minimal discussion of contradictory evidence clarify what this review adds beyond prior reviews.”* |
| --- | --- |
| **RESPONSE** | We strengthened critical synthesis in the Discussion by (i) explicitly contrasting competing governance perspectives (centralized command vs. polycentric/decentralized governance; “more coordination platforms” vs. “consolidated authority” positions); (ii) flagging where the evidence diverges or is contradictory (e.g., centralized arrangements reported as enabling rapid mobilization yet also as constraining local responsiveness; decentralization reported as improving responsiveness yet also as fragmenting accountability where unfunded); and (iii) adding a sentence stating this review’s specific contribution beyond prior reviews namely the integration of governance, coordination, and accountability as a single mapped system with an explicit evidence-type differentiation and conceptual framework. |
| **REVISION** | Discussion: added a critical-synthesis paragraph and “This review adds to prior, more narrowly scoped reviews by mapping governance, coordination, and accountability as an interconnected system rather than as separate domains, by differentiating evidence types, and by offering an integrated descriptive framework (Figure 2).” Contradictory-evidence signposting added under Themes 1 and the centralization–decentralization subsection. |

| **COMMENT** | *Reviewer 3 (and Editor): protocol registration; Reviewer 2/limitations: descriptive nature and definitional variation affecting comparability.* |
| --- | --- |
| **RESPONSE** | We acknowledge the protocol was not registered and now state this plainly as a limitation. We also expanded the limitations to reflect that the descriptive nature of many included studies and the heterogeneous definitions of governance, coordination, and accountability constrain comparability across the evidence base. |
| **REVISION** | Methods retains “No protocol was registered” and Limitations now adds “the review protocol was not registered; and because many included studies were descriptive and defined governance, coordination, and accountability heterogeneously, comparability across sources is limited and the mapped patterns should be read as descriptive rather than evaluative.” |

| **COMMENT** | *Reviewer 3 / Minor: terminology consistency (“multi sector” vs “multisector”); time-frame inconsistency (2000–2020 vs 2000–2025); typographical issues.* |
| --- | --- |
| **RESPONSE** | Corrected throughout. We standardised on “multisector” (and “multisectoral”), reconciled the timeframe to the actual search window 2000–2025 wherever the descriptive “2000–2020” had been used in error, and corrected residual typographical and spacing inconsistencies. |
| **REVISION** | Global find-and-replace “multi sector” → “multisector”; “2000 and 2020” → “2000 and 2025” in Results; spacing/typographic fixes throughout (shown in green). |

**Part E Journal requirements**

| **COMMENT** | *(1) Provide separate figure files in .tif or .eps.* |
| --- | --- |
| **RESPONSE** | Both figures (Figure 1, PRISMA-ScR flow diagram; new Figure 2, conceptual framework) are provided as separate high-resolution .tif/.eps files meeting the journal’s specifications. |

| **COMMENT** | *(2) Add a full list of Supporting Information legends after the references.* |
| --- | --- |
| **RESPONSE** | Added. A “Supporting Information” legends list now appears after the References. |
| **REVISION** | Added: “S1 Table. PRISMA-ScR checklist. S2 Table. Full database search strategies and retrieval counts. S3 Table. Data-extraction template and variable definitions. S4 Table. Complete coding framework/codebook. S5 Table. Characteristics of included sources with evidence-type classification.” |

| **COMMENT** | *(3) Confirm the Data Availability Statement / minimal data set.* |
| --- | --- |
| **RESPONSE** | We confirm that no primary data were generated. The “minimal data set” required to replicate the synthesis the full list of included sources, the extracted data matrix, and the coding framework is provided in the Supporting Information (S2–S4). We have retained and lightly revised the Data Availability Statement accordingly. |
| **REVISION** | Data Availability: “All data underlying the findings are within the manuscript and its Supporting Information files (S1–S5), including the complete list of included sources, the extracted data matrix, and the coding framework. No primary data were collected or generated.” |

We are grateful to the editor and reviewers for comments that have materially improved the rigour, transparency, and clarity of the manuscript. We believe the revised version now fully reflects the descriptive scope of a scoping review while offering an integrated, policy-relevant synthesis, and we hope it is suitable for publication in PLOS Global Public Health.

On behalf of all authors,

**Sileshi Demelash Sasie**

*Ethiopian Public Health Institute, Addis Ababa, Ethiopia*
