# Supplementary material for: Governance, coordination, and accountability in public health emergency preparedness and response: A scoping review
Source: PLOS Glob Public Health. 2026 Sep 18;6(9):e0006719. doi: 10.1371/journal.pgph.0006719 (PMC13600703; doi:10.1371/journal.pgph.0006719)
Supplement: Peer review history

# Peer review: Decision Letter 0

**DOI:** 10.1371/journal.pgph.0006719.r001

**Authors:** Catherine Grant

11 May 2026

PGPH-D-26-00735

Governance, Coordination, and Accountability in Public Health Emergency Preparedness and Response: A Scoping Review

PLOS Global Public Health

Dear Dr. Sasie,

Thank you for submitting your manuscript to PLOS Global Public Health. After careful consideration, we feel that it has merit but does not fully meet PLOS Global Public Health’s publication criteria as it currently stands. Therefore, we invite you to submit a revised version of the manuscript that addresses the points raised during the review process.

**Editor Comments**

Thank you for submitting this scoping review to PLOS Global Public Health. The manuscript addresses a timely and policy-relevant topic, and all three reviewers recognise its merits. The manuscript is recommended for **minor revision** . Please address the following points before resubmission.

**Required for acceptance**

*Analytical framing and conclusions*

The most significant issue raised — particularly by Reviewer 3, but consistent with concerns from Reviewer 1 — is that the manuscript's conclusions at times imply causal or evaluative claims (e.g., that certain governance factors "shape" or determine preparedness outcomes) that are not analytically justified within a scoping review methodology. You are required to either: (a) reframe conclusions to accurately reflect the descriptive and mapping scope of the review, using language such as "associated with," "reported alongside," or "identified in relation to"; or (b) provide explicit analytical justification if causal inference is intended. The discussion should interpret findings rather than restate results — please revise accordingly to reduce redundancy between the Results and Discussion sections.

*Methodological transparency*

Reviewers 2 and 3 both flag insufficient detail on the data extraction and coding process. You are required to provide, as supplementary materials: the full data extraction template, the coding framework or codebook, and the complete search strings used. This is essential for reproducibility and meets PLOS Global Public Health's standards for methodological rigour.

*Evidence differentiation*

Reviewer 3 raises a valid concern, also implicit in Reviewer 1's request for tangible examples, that the manuscript treats conceptual papers, empirical studies, and grey literature as equivalent sources without distinguishing the strength or type of evidence behind each finding. You are required to clearly differentiate evidence types across themes — for example, by indicating in the synthesis or tables whether claims are supported by empirical data, conceptual argument, or policy documentation — and to explicitly acknowledge the limitations of including grey literature alongside peer-reviewed sources.

*Thematic clarity*

Reviewer 2 notes that Theme 5 (centralisation and decentralisation) overlaps substantially with Theme 1 (governance architecture). You are required to either clarify the analytical distinction between these themes in the text, or merge them with appropriate restructuring.

**Recommended (not required for acceptance, but would strengthen the manuscript)**

*Conceptual or analytical framework*

Reviewer 3 recommends developing a conceptual model or diagram that integrates governance structures, coordination mechanisms, and accountability into a unified framework. Reviewer 1 similarly requests a figure on page 5 (line 102) to support comprehension of the analytical structure. The editors support this recommendation: a visual framework summarising relationships between key constructs would substantially improve the manuscript's contribution and accessibility. This is recommended but not required.

*Country/regional specificity and examples*

Reviewer 1 requests tangible country-level examples for each theme (pages 14–21), a clarification of which countries have yet to implement certain frameworks (page 3, line 66), and explanation of what the patterns at line 434 (page 23) mean in practical terms. Reviewer 2 recommends streamlining the Ethiopia section to avoid imbalance with the global discussion. The editors recommend the authors address these points to improve concreteness and global balance, though authors should exercise judgement on how to do so without disproportionately expanding the manuscript.

*Specific queries from Reviewer 1*

Please respond to the following in either the revised text or the rebuttal letter: (a) the apparent decline in publications 2021–2024 despite COVID-19 (page 14, line 270) — an explanation or acknowledgement of this trend is expected; (b) the meaning and purpose of Table 3 (page 20) — if it cannot be made self-explanatory with revision, consider whether it adds sufficient value to retain; (c) the point about enforceability of policies (page 27, line 531) — brief examples or references would strengthen this.

Please submit your revised manuscript by Jun 10 2026 11:59PM. If you will need more time than this to complete your revisions, please reply to this message or contact the journal office at globalpubhealth@plos.org. When you're ready to submit your revision, log on to https://www.editorialmanager.com/pgph/ and select the 'Submissions Needing Revision' folder to locate your manuscript file.

Please include the following items when submitting your revised manuscript:

- A letter that responds to each point raised by the editor and reviewer(s). You should upload this letter as a separate file labeled 'Response to Reviewers'.
- A marked-up copy of your manuscript that highlights changes made to the original version. You should upload this as a separate file labeled 'Revised Manuscript with Track Changes'.
- An unmarked version of your revised paper without tracked changes. You should upload this as a separate file labeled 'Manuscript'.

Guidelines for resubmitting your figure files are available below the reviewer comments at the end of this letter.

As the corresponding author, your ORCID iD is verified in the submission system and will appear in the published article. PLOS supports the use of ORCID, and we encourage all coauthors to register for an ORCID iD and use it as well. Please encourage your coauthors to verify their ORCID iD within the submission system before final acceptance, as unverified ORCID iDs will not appear in the published article. *Only* the individual author can complete the verification step; PLOS staff *cannot*  verify ORCID iDs on behalf of authors.

We look forward to receiving your revised manuscript.

Kind regards,

Catherine Grant

Academic Editor

PLOS Global Public Health

Journal Requirements:

1. Please provide separate figure files in .tif or .eps format.

For more information about figure files please see our guidelines:  LINK

https://journals.plos.org/globalpublichealth/s/figures

https://journals.plos.org/globalpublichealth/s/figures#loc-file-requirements

2. We have noticed that you have uploaded Supporting Information files, but you have not included a list of legends. Please add a full list of legends for your Supporting Information files after the references list.

3. We note that your Data Availability Statement is currently as follows: "All data underlying the findings of this study are fully available without restriction. This scoping review is based entirely on published peer-reviewed articles and publicly accessible grey-literature sources, all of which are cited within the manuscript and its supplementary materials. No primary data were collected, generated, or analysed for this study."

Please confirm at this time whether or not your submission contains all raw data required to replicate the results of your study. Authors must share the “minimal data set” for their submission. PLOS defines the minimal data set to consist of the data required to replicate all study findings reported in the article, as well as related metadata and methods (https://journals.plos.org/plosone/s/data-availability#loc-minimal-data-set-definition).

For example, authors should submit the following data:

- The values behind the means, standard deviations and other measures reported;

- The values used to build graphs;

- The points extracted from images for analysis.

Authors do not need to submit their entire data set if only a portion of the data was used in the reported study.

If your submission does not contain these data, please either upload them as Supporting Information files or deposit them to a stable, public repository and provide us with the relevant URLs, DOIs, or accession numbers. For a list of recommended repositories, please see https://journals.plos.org/plosone/s/recommended-repositories.

If there are ethical or legal restrictions on sharing a de-identified data set, please explain them in detail (e.g., data contain potentially sensitive information, data are owned by a third-party organization, etc.) and who has imposed them (e.g., an ethics committee). Please also provide contact information for a data access committee, ethics committee, or other institutional body to which data requests may be sent. If data are owned by a third party, please indicate how others may request data access.

If the reviewer comments include a recommendation to cite specific previously published works, please review and evaluate these publications to determine whether they are relevant and should be cited. There is no requirement to cite these works unless the editor has indicated otherwise.

Please review your reference list to ensure that it is complete and correct. If you have cited papers that have been retracted, please include the rationale for doing so in the manuscript text, or remove these references and replace them with relevant current references. Any changes to the reference list should be mentioned in the rebuttal letter that accompanies your revised manuscript. If you need to cite a retracted article, indicate the article’s retracted status in the References list and also include a citation and full reference for the retraction notice.

Additional Editor Comments (if provided):

[Note: HTML markup is below. Please do not edit.]

Reviewers' comments:

Reviewer's Responses to Questions

**Comments to the Author**

1. Does this manuscript meet PLOS Global Public Health’s publication criteria?

Reviewer #1: Yes

Reviewer #2: Yes

Reviewer #3: Yes

**********

2. Has the statistical analysis been performed appropriately and rigorously?

Reviewer #1: N/A

Reviewer #2: Yes

Reviewer #3: Yes

**********

3. Have the authors made all data underlying the findings in their manuscript fully available (please refer to the Data Availability Statement at the start of the manuscript PDF file)?

The PLOS Data policy

Reviewer #1: Yes

Reviewer #2: Yes

Reviewer #3: Yes

**********

4. Is the manuscript presented in an intelligible fashion and written in standard English?

Reviewer #1: Yes

Reviewer #2: Yes

Reviewer #3: Yes

**********

Reviewer #1: This is an excellent paper and I appreciated the authors laying out clearly their methods, the data sources and their findings. My comments are largely minor.

On page 3, line 66, the authors say 'many countries have yet'- could they specify what percentage and where? Which are these countries?

On page 5, line 102- it would be helpful to have a figure to explain the text better.

On page 14, line 270, the authors note fewer publications from 2021-2024- this seemed surprising to me given COVID- do they have any explanation for this trend?

Page 14/15 onwards: I think the author need to provide an example for each theme- like a tangible examples of what this theme means from a certain country context. Otherwise it feels too amorphous.

Page 20, Table 3- I didn't understand this table. What is it trying to say and why were these publications pulled out?

Page 21, Table 4, I think it would be helpful to have examples of each of the factors to explain what this exactly means practically within a national context

Page 23, line 434, Are the authors saying that political leadership and resources like money don't matter? I wasn't clear what this line is saying about the patterns and findings

Page 27, line 531, the authors make an important point about policies needing to be enforceable- do they have any thoughts or examples of how to do this?

Reviewer #2: PLOS Global Public Health Governance, Coordination, and Accountability in Public Health Emergency Preparedness and Response: A Scoping Review

Manuscript Number PGPH-D-26-00735

Reviewer Comments

This is a strong and carefully conducted study that addresses an important gap in understanding governance arrangements for public health emergency preparedness and response. The analysis is well grounded in both empirical evidence and relevant frameworks, and the use of multiple data sources adds depth and credibility to the findings. The manuscript is timely, relevant, and offers practical insights for policymakers and health system actors. Overall, the paper is well written and makes a meaningful contribution. If the points outlined below are considered, the manuscript would be further strengthened and its clarity and overall impact improved.

1. Background

The background is comprehensive and clearly written. However, the section on Ethiopia appears relatively detailed compared to the broader global discussion. It may be helpful to present Ethiopia more briefly as an illustrative example or to streamline this section so that the global focus is maintained more consistently throughout.

2. Methods

The methods are appropriate and generally clear. It would be helpful to briefly clarify whether the search strategy itself was independently verified by a second reviewer, in addition to the screening process that is already well described.

3. Results

Theme 5 on centralization and decentralization appears to overlap with Theme 1 on governance architecture. It may be useful to further clarify the distinction between these themes or to consider merging them so that the structure of the results is easier to follow.

4. Limitations

The limitations are clearly presented and appropriate for a scoping review. It may, however, be useful to more explicitly reflect on how the descriptive nature of the included studies, together with variation in how governance, coordination, and accountability are defined across contexts, may influence comparability of findings.

Overall Assessment

This is a well-prepared and thoughtful manuscript. With minor refinement, it is suitable for publication and will be useful for both national and global audiences working on public health emergency preparedness.

This is a solid and credible study with clear relevance to current global health priorities. The analysis is sound, and the findings are meaningful. I would recommend acceptance after minor revision.

Reviewer #3: Reviewer Report

Manuscript Title: Governance, Coordination, and Accountability in Public Health Emergency Preparedness and Response: A Scoping Review

Recommendation: Minor Revision

Summary of the Manuscript

This manuscript presents a scoping review of 77 studies (2000–2025) examining governance, coordination, and accountability in public health emergency preparedness and response. Using PRISMA-ScR methodology, the authors identify key themes related to institutional arrangements, coordination mechanisms, and systemic challenges, with emphasis on multisector collaboration and policy frameworks.

The topic is highly relevant, particularly for low- and middle-income countries, and the manuscript compiles a substantial body of literature. However, important methodological, analytical, and interpretive limitations weaken its contribution in its current form.

Some Comments

1. Clarity and Strength of Main Claims

The manuscript argues that:

• Governance, coordination, and accountability systems are central determinants of preparedness.

• Fragmentation, weak accountability, and unclear mandates undermine emergency response.

• Strong systems correlate with legal clarity, coordination bodies, and performance monitoring.

Concerns

• The claims are broad and largely confirm existing consensus, rather than offering new insights.

• There is no clear differentiation between descriptive mapping and causal inference.

• The conclusion suggests influence (“preparedness is shaped by…”) without analytical justification beyond thematic synthesis.

Recommendation

• Reframe conclusions to reflect descriptive scope rather than causal interpretation, OR

• Strengthen analytical framing to justify causal language.

2. Engagement with Existing Literature

Strengths

• Wide coverage of global and regional literature

• Inclusion of both peer-reviewed and grey literature

• Relevant references to international frameworks (e.g., IHR)

Limitations

• Literature is summarized rather than critically synthesized

• Limited engagement with:

Competing governance theories

Differences across political or health system contexts

• Minimal discussion of contradictory evidence or debates

Recommendation

• Introduce critical comparison across studies

• Highlight where evidence diverges or is inconsistent

• Clarify what this review adds beyond prior reviews

3. Methodological Rigor and Transparency

Strengths

• PRISMA-ScR framework followed ✔

• Clear eligibility criteria ✔

• Dual screening with inter-rater reliability (kappa = 0.82) ✔

Limitations

• No formal quality appraisal (acceptable for scoping, but limits interpretation)

• Insufficient detail on:

Data extraction framework (not fully presented)

Coding process

Thematic development

Critical Issue

The manuscript explicitly states:

Better to clearly state causal or effectiveness assessment was conducted

Yet conclusions imply:

• Relative importance of governance factors

• Policy effectiveness

Recommendation

• Clarify analytical boundaries

• Provide supplementary materials:

Data extraction template

Coding framework

Example coded data

4. Data and Evidence Supporting Claims

Findings

• Themes are clearly presented and logically structured.

• Evidence is heterogeneous (conceptual, empirical, policy reports).

Concerns

• Provide cleared distinction between:

Strong vs weak evidence

Empirical vs conceptual studies

• No weighting or hierarchy of evidence

Implication

• Conclusions risk overgeneralization.

Recommendation

• Clearly distinguish:

Types of evidence

Strength of support

• Avoid language implying effectiveness or causality

5. Use of Grey Literature

Strength

• Inclusion adds policy relevance

Concern

• Grey literature is treated equally to peer-reviewed studies

• No discussion of:

Potential bias

Differences in methodological rigor

Recommendation

• Explicitly acknowledge limitations of grey literature

• Consider separating synthesis or interpretation

6. Need for Conceptual or Analytical Framework

Currently, the manuscript:

• Identifies themes

• Describes relationships

But does not

• Integrate findings into a unified framework

Recommendation

Develop a:

• Conceptual model of governance-accountability-preparedness

• Or analytical framework showing relationships between:

Governance structures

Coordination mechanisms

Outcomes

This would significantly strengthen contribution.

7. Reproducibility and Data Availability

Current Status

• Authors state all data are publicly available

Limitations

• No publicly available dataset of:

Included studies

Extracted variables

• Search strategy not fully reproducible within manuscript

Recommendation

• Provide:

Full search strings

Extraction dataset (as supplementary file)

8. Reporting Guidelines Compliance

• PRISMA-ScR: largely followed ✔

• Arksey & O’Malley framework: applied ✔

Minor Gaps

• No protocol registration

• Limited reporting of:

Data charting process

Analytical decision-making

9. Writing, Structure, and Accessibility

Strengths

• Logical structure

• Clear sectioning

• Accessible language for multidisciplinary audience

Weaknesses

• Repetition of key terms without added analytical depth

• Some sections overly descriptive

• Discussion occasionally reiterates results rather than interpreting them

Recommendation

• Reduce redundancy

• Strengthen analytical interpretation in Discussion

Minor Comments

Ensure consistent terminology (e.g., “multi sector” vs “multisector”)

Clarify time frame inconsistencies (some sections report 2000–2020 vs 2000–2025)

Improve clarity of figures/tables (not fully assessable in text extract)

Remove minor typographical inconsistencies

Responses to Specific PLOS Criteria

Criterion Assessment

Main claims & significance: Major contribution; needs some analytical positioning

Placement in literature: Adequate

Data support claims: Partially supported; risk of over interpretation

Protocol adherence: Good adherence

Need for protocols/algorithms: Yes – conceptual framework recommended

Suitability for publication: Yes, after minor revision

Data availability: Acceptable

Guideline compliance: Generally compliant

Method reproducibility: Moderate; needs more transparency

Software availability: Not applicable

Clarity and accessibility: Good, but repetitive

Final Recommendation

Minor Revision Required

Key Actions for Authors (Priority Order)

1. Align conclusions with methodology: Avoid causal language unless justified

2. Add conceptual/analytical framework: Move beyond descriptive synthesis

3. Improve methodological transparency: Provide extraction and coding details

4. Differentiate evidence types: Avoid treating all sources equally

5. Strengthen discussion: Interpret, not just restate findings

Closing Assessment

This manuscript addresses very important and policy-relevant topic, and the dataset is valuable. If some improvements made, it helps for policy makers and its contribution to the field is vital.

With substantial revision—particularly strengthening analytical depth and methodological clarity—the paper could reach the standard expected for publication.

**********

what does this mean?). If published, this will include your full peer review and any attached files.

**Do you want your identity to be public for this peer review?** If you choose “no”, your identity will remain anonymous but your review may still be made public.

For information about this choice, including consent withdrawal, please see our Privacy Policy

Reviewer #1: No

Reviewer #2: **Yes:**  Yohannes Mulugeta Demmu

Reviewer #3: No

**********

[NOTE: If reviewer comments were submitted as an attachment file, they will be attached to this email and accessible via the submission site. Please log into your account, locate the manuscript record, and check for the action link "View Attachments". If this link does not appear, there are no attachment files.]

Attachment Submitted filename: Reviewer Report.docx

Attachment Submitted filename: PGPH-D-26-00735.pdf

# Author comment: Author response to Decision Letter 1

**DOI:** 10.1371/journal.pgph.0006719.r002

10 Jun 2026

Attachment Submitted filename: Response_to_Reviewers.docx

# Peer review: Decision Letter 1

**DOI:** 10.1371/journal.pgph.0006719.r003

**Authors:** Julia Robinson

4 Sep 2026

Governance, Coordination, and Accountability in Public Health Emergency Preparedness and Response: A Scoping Review

PGPH-D-26-00735R1

Dear Dr Sasie,

We are pleased to inform you that your manuscript 'Governance, Coordination, and Accountability in Public Health Emergency Preparedness and Response: A Scoping Review' has been provisionally accepted for publication in PLOS Global Public Health.

Before your manuscript can be formally accepted you will need to complete some formatting changes, which you will receive in a follow up email. A member of our team will be in touch with a set of requests.

Please note that your manuscript will not be scheduled for publication until you have made the required changes, so a swift response is appreciated.

IMPORTANT: The editorial review process is now complete. PLOS will only permit corrections to spelling, formatting or significant scientific errors from this point onwards. Requests for major changes, or any which affect the scientific understanding of your work, will cause delays to the publication date of your manuscript.

If your institution or institutions have a press office, please notify them about your upcoming paper to help maximize its impact. If they'll be preparing press materials, please inform our press team as soon as possible -- no later than 48 hours after receiving the formal acceptance. Your manuscript will remain under strict press embargo until 2 pm Eastern Time on the date of publication. For more information, please contact globalpubhealth@plos.org.

Thank you again for supporting Open Access publishing; we are looking forward to publishing your work in PLOS Global Public Health.

Best regards,

Julia Robinson

Executive Editor

PLOS Global Public Health

***********************************************************

Reviewer Comments (if any, and for reference):

Reviewer's Responses to Questions

**Comments to the Author**

Reviewer #3: All comments have been addressed

**********

publication criteria?

Reviewer #3: Yes

**********

3. Has the statistical analysis been performed appropriately and rigorously?

Reviewer #3: Yes

**********

4. Have the authors made all data underlying the findings in their manuscript fully available (please refer to the Data Availability Statement at the start of the manuscript PDF file)?

The PLOS Data policy

Reviewer #3: Yes

**********

5. Is the manuscript presented in an intelligible fashion and written in standard English?

Reviewer #3: Yes

**********

Reviewer #3: I thank you the authors. I'm very satisfied with the responses.

**********

what does this mean?). If published, this will include your full peer review and any attached files.

**Do you want your identity to be public for this peer review?** If you choose “no”, your identity will remain anonymous but your review may still be made public.

For information about this choice, including consent withdrawal, please see our Privacy Policy

Reviewer #3: **Yes:**  Ephrem Geja Gechere

**********
